# Transcriptomic Profiling in Fins of Atlantic Salmon Parasitized with Sea Lice: Evidence for an Early Imbalance Between Chalimus-Induced Immunomodulation and the Host’s Defense Response

**DOI:** 10.3390/ijms21072417

**Published:** 2020-03-31

**Authors:** Navaneethaiyer Umasuthan, Xi Xue, Albert Caballero-Solares, Surendra Kumar, Jillian D. Westcott, Zhiyu Chen, Mark D. Fast, Stanko Skugor, Barbara F. Nowak, Richard G. Taylor, Matthew L. Rise

**Affiliations:** 1 Department of Ocean Sciences, Memorial University of Newfoundland, St. John’s, NL A1C 5S7, Canada; xi.xue@mun.ca (X.X.); acaballeroso@mun.ca (A.C.-S.); surendrak@mun.ca (S.K.); 2 Fisheries and Marine Institute, Memorial University of Newfoundland, P.O. Box 4920, St. John’s, NL A1C 5R3, Canada; jillian.westcott@mi.mun.ca (J.D.W.); zc5118@mun.ca (Z.C.); 3 Department of Pathology and Microbiology, Atlantic Veterinary College, University of Prince Edward Island, Charlottetown, PE C1A 4P3, Canada; mfast@upei.ca; 4 Cargill Aqua Nutrition, Cargill, Sea Lice Research Center (SLRC), Hanaveien 17, 4327 Sandnes, Norway; stanko_skugor@cargill.com; 5 Institute of Marine and Antarctic Studies, University of Tasmania, Locked Bag 1370, Launceston 7250, TAS, Australia; b.nowak@utas.edu.au; 6 Cargill Animal Nutrition, 10383 165th Avenue NW, Elk River, MN 55330, USA; richard_taylor@cargill.com

**Keywords:** *Lepeophtheirus salmonis*, chalimus, *Salmo salar*, fin transcriptome, immunomodulation, anti-sea lice response, 44K microarray, immunogenomics

## Abstract

Parasitic sea lice (e.g., *Lepeophtheirus salmonis*) cause costly outbreaks in salmon farming. Molecular insights into parasite-induced host responses will provide the basis for improved management strategies. We investigated the early transcriptomic responses in pelvic fins of Atlantic salmon parasitized with chalimus I stage sea lice. Fin samples collected from non-infected (i.e., pre-infected) control (PRE) and at chalimus-attachment sites (ATT) and adjacent to chalimus-attachment sites (ADJ) from infected fish were used in profiling global gene expression using 44K microarrays. We identified 6568 differentially expressed probes (DEPs, FDR < 5%) that included 1928 shared DEPs between ATT and ADJ compared to PRE. The ATT versus ADJ comparison revealed 90 DEPs, all of which were upregulated in ATT samples. Gene ontology/pathway term network analyses revealed profound changes in physiological processes, including extracellular matrix (ECM) degradation, tissue repair/remodeling and wound healing, immunity and defense, chemotaxis and signaling, antiviral response, and redox homeostasis in infected fins. The QPCR analysis of 37 microarray-identified transcripts representing these functional themes served to confirm the microarray results with a significant positive correlation (*p* < 0.0001). Most immune/defense-relevant transcripts were downregulated in both ATT and ADJ sites compared to PRE, suggesting that chalimus exerts immunosuppressive effects in the salmon’s fins. The comparison between ATT and ADJ sites demonstrated the upregulation of a suite of immune-relevant transcripts, evidencing the salmon’s attempt to mount an anti-lice response. We hypothesize that an imbalance between immunomodulation caused by chalimus during the early phase of infection and weak defense response manifested by Atlantic salmon makes it a susceptible host for *L. salmonis*.

## 1. Introduction

Ectoparasitic arthropods are the most prominent aquatic pest threat to the fishery and fish-farming industries worldwide [1,2]. Sea lice are naturally occurring Caligid crustacean parasites that infect both wild and farmed salmonids, causing disease and, in some extreme cases, mortalities, and are responsible for escalating economic losses estimated at hundreds of million dollars (USD) annually [3]. Two sea lice genera devastating aquafarming of various salmonids have provoked a global concern: while *Lepeophtheirus* (i.e., *L. salmonis*) mainly infects salmonids in the Northern hemisphere, the prevalence of *Caligus* (several spp. including, *C. clemensi*, *C. elongatus*, and *C. rogercresseyi*) have been reported from oceans of both the Northern and Southern hemispheres (reviewed in References [2,4]).

*L. salmonis* (Krøyer, 1838), commonly known as the salmon louse, exhibits a narrow host range compared to that of the aforementioned *Caligus* spp. and parasitizes genera of salmonids, including *Salmo*, *Oncorhynchus*, and *Salvelinus.* The host susceptibility and resulting physiological and pathological consequences caused by the infection depend on factors associated with both host (e.g., species age, reproductive stage, and presence of other infections) and parasite (e.g., host preference and developmental stage) [2,5]. Atlantic salmon (*Salmo salar*) is among the most intensively farmed finfish species [6,7], but is also more vulnerable to *L. salmonis* infection [2,4] than some species of Pacific salmon (e.g., coho (*O. kisutch*) and pink (*O. gorbuscha*) salmon) [8,9]. This is thought to be primarily due to its weaker cellular response to the louse (i.e., the attached chalimus life stages), such as limited epithelial hyperplasia and inflammation [10], compared to other salmonids.

The life cycle of *L. salmonis* comprises eight distinct developmental stages: sea lice disperse as non-feeding planktonic nauplii (2 stages), which molt to infective copepodids (1 stage) that attach to the host and develop to chalimi (2 stages), which then attach to host skin or fins to feed. Subsequent developmental stages include mobile pre-adults (2 stages) and adults (1 stage) that feed on mucus, and blood in the skin (head and dorsal). This causes greater damage, resulting in lesions and skin erosion due to their larger size, mobility, and aggressive feeding nature [10,11]. The progressive development of lesions, open wounds, and extensive tissue damage can cause chronic stress coupled with impaired growth [10]. Additionally, impacts of sea lice on the host immune system result in greater susceptibility to secondary bacterial [12] and viral [13] infections. Recently, there has been concern regarding the potential for sea lice to spread from salmon farms to wild salmonid populations [2,5].

The success and sustainability of the Atlantic salmon farming industry, and the welfare of wild salmon populations, are dependent on effective disease prevention, control, and health management regimes. Many such strategies are currently in use or being developed, including chemotherapeutants (e.g., ‘in-feed’ immunostimulants [14] and probiotics [15] or in situ ‘baths’ with parasiticides [16]), vaccination [17], and cleaner fish [2,5,18,19]. Since each of these sea lice control strategies has advantages and disadvantages, an Integrated Pest Management (IPM) approach has been employed [20]. Recently, in addition to IPM, research has focused more on ‘green-technology’ to be incorporated into sea lice management [21,22].

A comprehensive understanding of defense mechanisms of salmon against sea lice may provide the basis for devising novel approaches as tools for sea lice control. In this regard, improved knowledge of genes and pathways that respond to the sea lice would lead to the development of valuable biomarkers for new intervention strategies. Previous transcriptomic studies that have attempted to explore the salmon immune responses from a global gene expression perspective primarily focused on local responses in skin and systemic responses in head kidney and/or spleen [8,15,23,24,25,26]. In a comparative profiling of fin transcriptome in three salmonid species 6 days post-exposure to *L. salmonis*, Sutherland et al. [8] found 130 and 4 differentially expressed genes (DEGs) in chum (*O. keta*) and pink salmon, respectively; whereas, no DEGs were discovered in Atlantic salmon fins [8]. Consequently, the mechanisms related to local responses in fins, which are the initial and preferential settlement sites of chalimi, against the early phase of *L. salmonis* infection are not completely understood. The present study addressed this gap of knowledge in the salmon’s anti-parasitic immune response using a 44K consortium for Genomic Research on All Salmonids Project (cGRASP) microarray platform [27] which provides good coverage of the salmon transcriptome, together with real-time quantitative polymerase chain reaction (QPCR) for confirmation purposes. We have used this approach in our previous immune-relevant experiments for studying salmon immune transcriptome responses against viral mimic [28] and bacterial antigens [29]. We investigated the initial local host transcriptomic response in pelvic fin, the first dominant attachment sites of *L. salmonis* copepodids [30], of infected salmon by profiling the gene expression at attachment sites (ATT) and adjacent to chalimus-attachment sites (ADJ) compared to non-infected controls (PRE). Our study was aimed at depicting the modulation of host responses against *L. salmonis* infection, which enabled us to explore, in detail, specific genes, pathways, and networks associated with the interface of host–parasite interaction.

## 2. Results

### 2.1. Sea Lice Load in Salmon

In this study, we used a host–parasite (*S. salar*-*L. salmonis*) model to investigate the transcriptomic responses of the host to infection. Pelvic fin tissue was sampled from non-infected fish (PRE; 0 days post-infection (dpi); *n* = 11), and salmon were experimentally infected with *L. salmonis* copepodids. At 8 dpi, the number of chalimi on each infected salmon was counted prior to the excision of fin tips for sampling from chalimus-attachment (ATT) and adjacent (ADJ) sites of each fish (*n* = 12).

Lice burden was 51 ± 6.3 chalimi/fish [mean ± standard error (SE)]. Chalimi (8 dpi) preferentially attached to fins (75.8%; 38.8 ± 5.7) as compared with gills (14.1%; 7.3 ± 1.2) and other regions of skin (10.1%; 5.2 ± 0.9) (Supplementary Figure S1). The pre-adult lice count at the end of the infection trial (30 dpi) was similar (i.e., 56 ± 3.4 lice/fish) to that of the chalimi count (8 dpi).

### 2.2. Global Transcriptomic Changes in Response to Chalimus Attachment

In order to explore the transcriptional response in fin induced by the attached *L. salmonis* chalimi on pelvic fins during the infection (at 8 dpi), the differentially expressed probes (DEPs) between fins from PRE, ADJ, and ATT groups (*n* = 6 samples/group) were identified using a 44K cGRASP salmon microarray platform [27].

**Figure 1:**
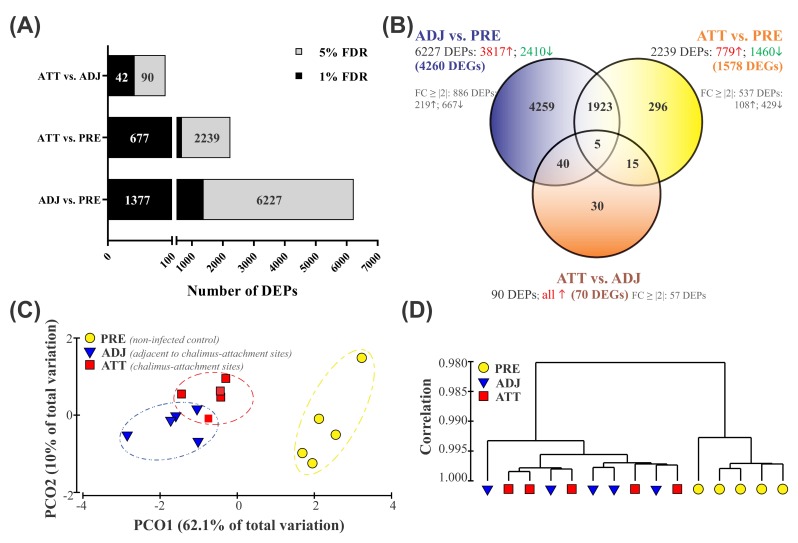
Overview of resulting global transcript expression profiles. (**A**) The global profile of differentially expressed probes (DEPs) identified by the Significance Analysis of Microarrays (SAM) algorithm using a modified *t*-test. Grey and black color bars (and the numbers on them) indicate the number of DEPs at false discovery rates (FDRs) of 5% and 1%, respectively. (**B**) Venn diagram showing the distribution of 6568 DEPs among three different comparisons of lice infection treatments. Numbers in red (↑) and green (↓) indicate up- and down-regulated DEPs, respectively. FC ≥ # indicates the number of DEPs with a fold-change value ≥ |2|. Detailed profiles of DEPs are available in Supplementary Files S1 and S2. (**C**,**D**) Principal coordinate analysis (PCoA) and hierarchical clustering analysis of fin samples from control (PRE) and sea lice-infected (ADJ and ATT) fish based on the expression dataset of DEPs (*n* = 6568). (**C**) PCoA of a resemblance matrix generated using Bray-Curtis similarity coefficients. (**D**) Complete linkage clustering was performed using Pearson correlation in the PRIMER 6.1.15 package. vs., versus.

The fin transcriptome was compared between sample groups using Significance Analysis of Microarrays (SAM) [31], and the profile of DEPs has been illustrated in Figure 1A,B. The current study identified a total of 6568 non-redundant DEPs when three different groups were compared in pairs (i.e., 6227 in ADJ compared to PRE (ADJ versus PRE), 2239 in ATT compared to PRE (ATT versus PRE), and 90 in ATT compared to ADJ (ATT versus ADJ)) at false discovery rate (FDR) < 5% (Figure 1A; Supplementary File S1). Of these DEPs, putative identities could be determined for 5373 DEPs by using BLASTn/x searches against NCBI nr/nt databases. A more stringent FDR cutoff of 1% yielded 1377, 677, and 42 chalimus-responsive transcripts for ADJ versus PRE, ATT versus PRE, and ATT versus ADJ comparisons, respectively (Figure 1A). All downstream analyses presented here are based on transcripts identified at FDR < 5%.

Among 6227 DEPs in the ADJ versus PRE comparison, 3817 probes were significantly upregulated and 2410 probes were significantly downregulated (Figure 1B). We identified 779 higher and 1460 lower expressed probes in the ATT versus PRE comparison, respectively. All 90 DEPs in the ATT versus ADJ comparison were significantly upregulated in ATT. The re-annotation of these probe lists provided information on DEGs for each comparison: 4260 DEGs for ADJ versus PRE, 1578 DEGs for ATT versus PRE, and 70 DEGs for ATT versus ADJ (Figure 1B). By taking the redundancy of the 44K platform into account, it was determined that 6568 DEPs represent 3571 DEGs with known putative identity. For complete information of these DEPs, including their identity based on BLAST annotation and fold-change (FC) values, refer to Supplementary Files S1 and S2.

A relatively clear clustering of PRE, ATT, and ADJ samples was evident from the principal coordinate analyses (PCoA) and hierarchical clustering analyses conducted based on gene expression data of the entire set of DEPs (6568; Figure 1C,D). PCO1 and PCO2 accounted for 62.1% and 10.0% of the variation among fish, respectively. Along with PCO1, animals were distinguished according to the treatment, with samples obtained from the PRE control clustered distantly from samples obtained from infected fish (ATT and ADJ; Figure 1C). Moreover, the grouping pattern in hierarchical clustering (Figure 1D) was also in good agreement with the PCoA results (Figure 1C), in which ATT and ADJ samples clustered closely with considerable overlap. These results suggest that ATT and ADJ samples (both collected from the same fish) may share an overall gene expression pattern.

**Figure 2:**
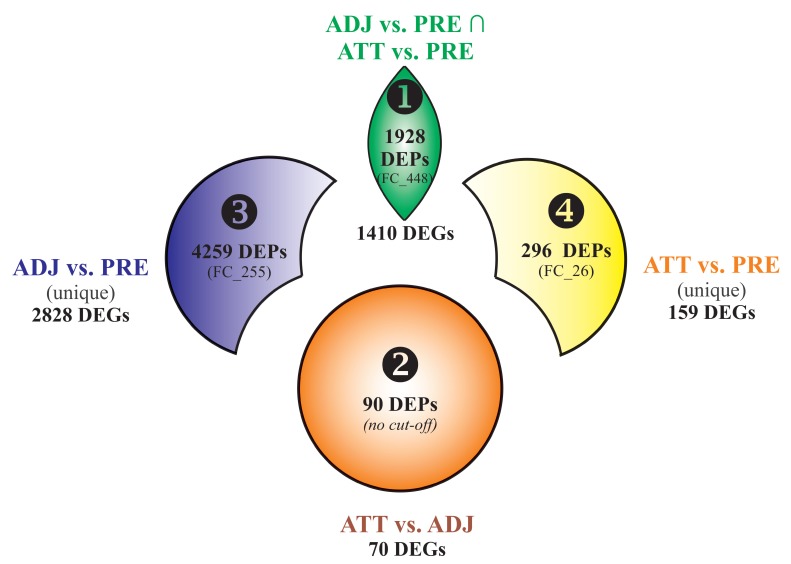
Venn diagram showing differentially expressed probes (DEPs) and/or genes (DEGs) among different segments considered in the Results and Discussion Sections. Each segment is assigned with a number (1–4). Bold numbers indicate the DEPs contributing to a particular segment. The number of DEGs corresponding to DEPs of each segment is shown. FC_# indicates the number of DEPs with a fold-change ≥ |2|. It should be noted that DEPs in segment 1 required to comply with the criteria of FC ≥ |2| in both comparisons ADJ versus PRE and ATT versus PRE. Only the features with FC ≥ |2| were subjected to Blast2GO enrichment analyses, except for segment 2 as this probe list was relatively shorter. Whereas, all the DEGs of each segment were used in gene ontology (GO)/pathway terms analyses using ClueGO. vs., versus.

Using Venn diagram analyses, the DEPs and DEGs were divided into 4 segments (Figure 2). Segment 1 comprised 1928 DEPs (1410 DEGs) overlapping between ADJ versus PRE and ATT versus PRE comparisons, and represented a robust set of chalimus-responsive fin transcripts. Segment 2 included 90 DEPs (70 DEGs) from the ATT versus ADJ list. Segments 3 and 4 comprised 4259 DEPs (2828 DEGs) and 296 DEPs (159 DEGs) that are exclusive to ADJ versus PRE and ATT versus PRE comparisons, respectively (Figure 2). The Results and Discussion Sections are presented based on analyses of these segments with a particular focus on segments 1 and 2.

### 2.3. Enriched Gene Ontology (GO) Terms Associated with DEPs

By increasing the FC cutoff to |2|, the DEPs were filtered to identify those likely to be biologically most relevant in terms of response to sea lice. This reduced the number of chalimus-responsive probes to 886 DEPs for ADJ versus PRE (219 upregulated and 667 downregulated), 537 for ATT versus PRE (108 upregulated and 429 downregulated), and 57 for ATT versus ADJ (all upregulated) (Figure 1B, Supplementary File S1). Gene ontology (GO) term analyses of DEPs were conducted by Blast2GO in two steps. In the first step, GO term distribution of different GO domains (biological process (BP), molecular function (MF), cellular component (CC)) was mapped. Secondly, Fisher’s Exact Test was conducted to study the enrichment of GO terms.

**Figure 3:**
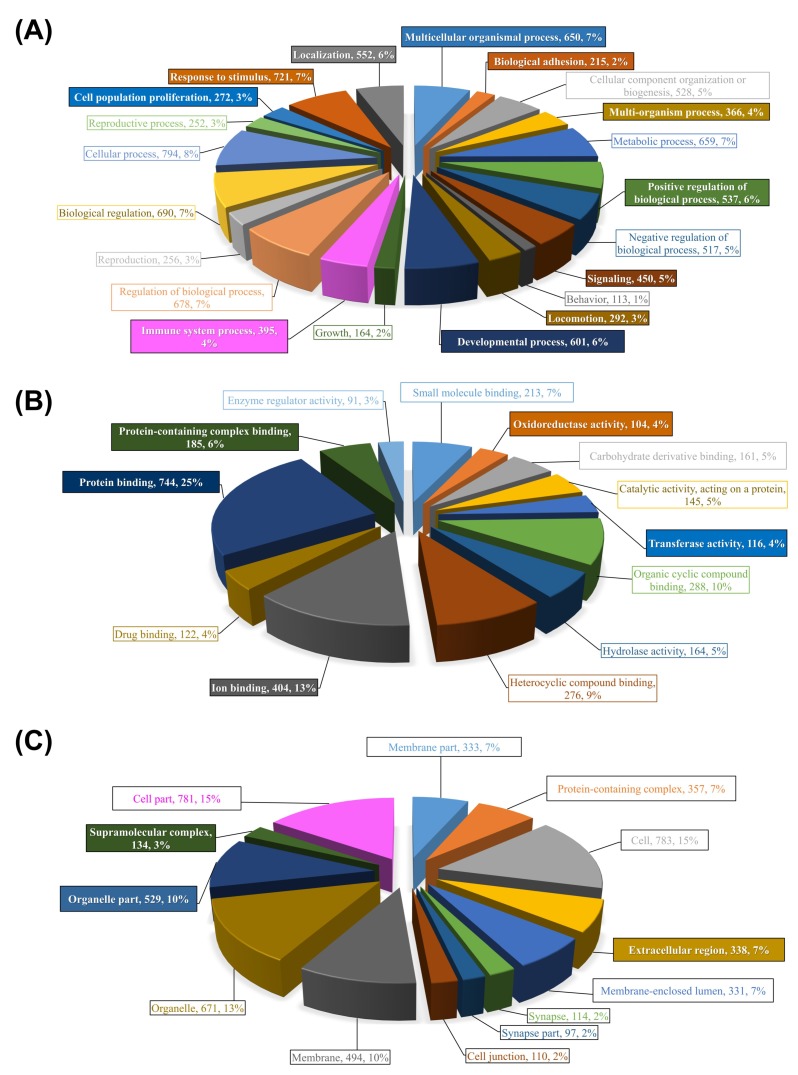
Summary of GO terms annotation for all DEPs responsive to sea lice infection and their enrichment analyses. Three different categories of GO terms are illustrated: (**A**) biological process (BP), (**B**) molecular function (MF), and (**C**) cellular component (CC). Non-redundant probes constituted from the union of probes with fold-change ≥ |2|from ADJ versus PRE and ATT versus PRE lists, and all DEPs from ATT vs. ADJ list (Figure 1B; Supplementary File S3; *n* = 1014 DEPs) were annotated using the Blast2GO package. Charts represent the distribution of GO terms, in which BP, MF, and CC are shown at GO levels 2, 3, and 2, respectively. Enrichment analyses were performed using the entire 44K array as reference (Fisher’s Exact Test, FDR < 0.01). Significantly enriched GO terms are boxed and color-filled. Complete lists of over-/under-represented GO terms and their statistics are available in Supplementary File S3.

Figure 3 illustrates the results of these two-step analyses for the entire list of chalimus-responsive DEPs. For these analyses, we used a list of 1014 DEPs constituted from the union of probes with FC ≥ |2|from ADJ versus PRE and ATT versus PRE lists, and all DEPs from ATT vs. ADJ list (Figure 1B; Supplementary File S3B). Among 21 level-2 GO BP terms, 11 were found to be enriched (e.g., response to stimulus (GO:0050896), signaling (GO:0023052), immune system process (GO:0002376), and biological adhesion (GO:0022610); Figure 3A). Five GO terms were enriched among 13 level-3 GO terms associated with MF (Figure 3B), in which many of them were related to ‘binding’ to different molecules (e.g., ion (GO:0043167) and protein (GO:0005515)), and oxidoreductase activity (GO:0016491). There were 3 level-2 GO CC terms that were enriched out of a total of 13 terms (e.g., ‘extracellular region’ (GO:0005576; Figure 3C)). It is worth noting that the majority of the GO (>90%) terms were over-represented (Supplementary File S3).

Detailed profiles of GO term distribution of BP and CC domains and their enrichment data for the different segments (i.e., 1, 2, and 3) of DEPs (Figure 2; FC ≥ |2|) by Blast2GO analysis are available in Supplementary Files S4–S6. Analysis of segment 4 yielded no enriched GO terms. Based on these profiles, multiple GO BP terms associated with stress and/or stimulus-response, and several immune processes were over-represented. Under the CC domain, the majority of the enriched terms were associated with extracellular matrix/region. Several GO MF terms related to O_2_ transport (e.g., heme/O_2_ binding) were also present in the enriched GO list (Supplementary File S4).

### 2.4. GO/Pathway Term Networks Associated with DEGs

**Figure 4:**
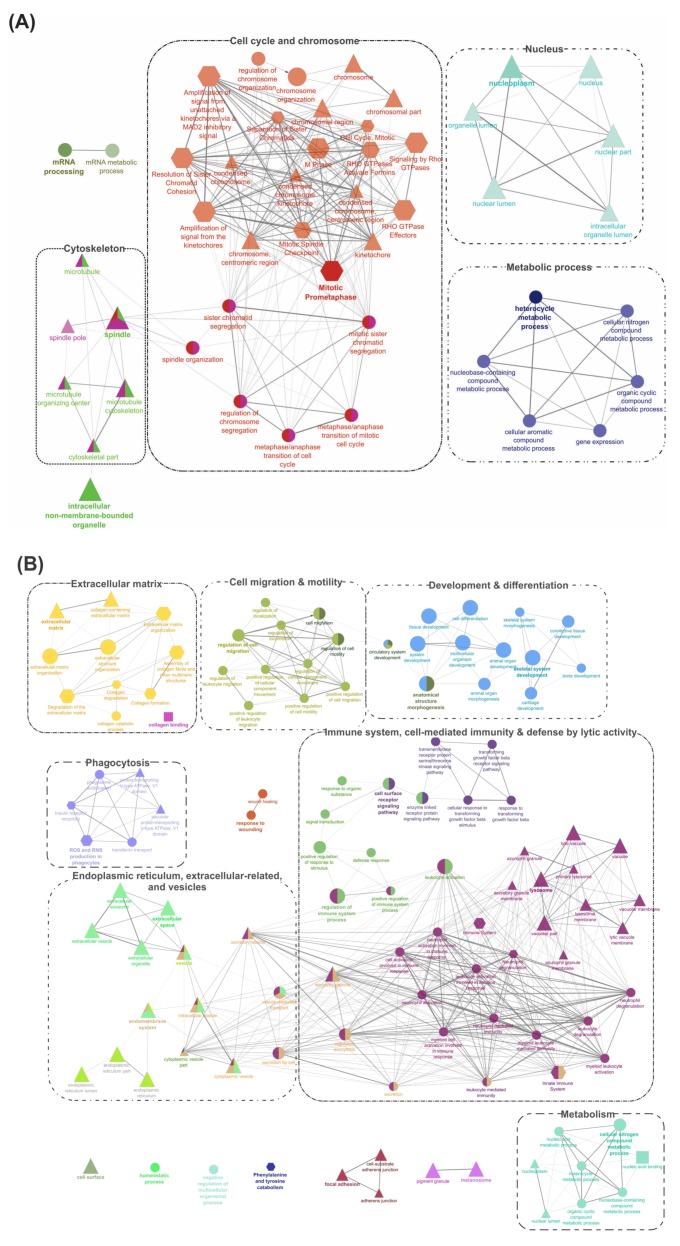
Gene-Ontology (GO) term enrichment and pathway term network analysis of DEGs shared by ADJ versus PRE and ATT versus PRE lists (1928 DEPs). (**A**) Upregulated DEGs (651 DEPs). (**B**) Downregulated DEGs (1277 DEPs). GO enrichment analysis was performed by the ClueGO [32] plugin in Cytoscape [33]. Two databases were used, including GO (BP, MF, and CC) and Reactome pathways for retrieving associated terms. Only the networks and pathways with *p* < 0.05 are illustrated. Functionally grouped networks with terms as nodes linked by edges based on their kappa score level (≥0.4) are shown. Related-GO terms are grouped and illustrated with distinct color and labeled with the same color. The node size represents the significance of term enrichment. Functionally related groups partially overlap. When a particular GO term is shared by two or more different GO cluster groups, the node is shown by multiple colors. The shape of the nodes indicates the source of the database from where a term was retrieved (ellipse, GO_BP; rectangle, GO_MF; triangle, GO_CC; Reactome, hexagon). The thickness of edges indicates the kappa score (strength of intra-connectivity between cluster groups). Related clusters are shown together within a dotted border and labeled with broad themes for discussion purposes. Refer to the Supplementary Files S7 and S8 for details and high-resolution images.

**Figure 5:**
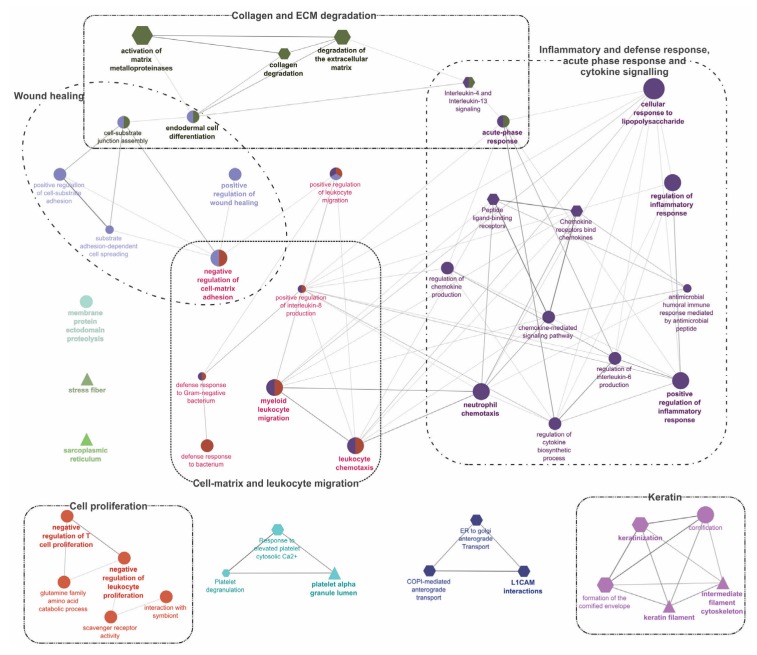
Gene-Ontology (GO) term enrichment and pathway term network analysis of DEGs in ATT versus ADJ list (90 DEPs). Refer to the caption of Figure 4 and Supplementary File S9 for additional details and the high-resolution image.

The ClueGO tool [32] was used to perform an integrated GO/pathway term network analysis by functionally organizing the enriched GO (BP, MF, and CC) and Reactome pathway terms. All four segments of DEGs (Figure 2) were separately analyzed for their up- and down-regulated transcripts, and the results are illustrated in Figure 4 and Figure 5, and Figures S2–S4. Detailed results of these analyses, including information about statistical parameters, clusters, and associated genes, are provided in Supplementary Files S7–S14.

Pathway enrichment results for up- and down-regulated DEGs in segment 1 (Figure 2; DEGs shared by ADJ versus PRE and ATT versus PRE) are provided in Figure 4 and Supplementary Files S7 and S8. DEGs representing upregulated transcripts of segment 1 formed four main clusters associated with ‘cytoskeleton’, ‘cell cycle and chromosome’, ‘nucleus’, and ‘metabolic process’ (Figure 4A). In contrast, GO pathway terms of downregulated transcripts in segment 1 were composed of seven clusters associated with ‘extracellular matrix (ECM)’, ‘cell migration and motility’, ‘development and differentiation’, ‘immune system, cell-mediated immunity and defense by lytic activity’, ‘endoplasmic reticulum (ER), extracellular-related and vesicles’, ‘phagocytosis’, and ‘metabolism’ (Figure 4B).

Segment 2 (Figure 2; DEGs in ATT versus ADJ list) presented six enriched GO term clusters associated with ‘cell-matrix and leukocyte migration’, ‘inflammatory and defense response, acute phase response (APR), and cytokine signaling’, ‘collagen and ECM degradation’, ‘wound healing’, ‘cell proliferation’, and ‘keratin’, among which, the first four clusters were interconnected (Figure 5 and Supplementary File S9). ‘Activation of matrix metalloproteinases’ (R-HSA:1592389; corrected *p*-value 1.86 × 10^−4^) was the most significant term and possessed the highest percentage of associated genes (19.05%) in this segment.

Enriched GO/pathway terms for segment 3 (Figure 2) that represented unique DEGs of ADJ versus PRE are shown in Supplementary Figure S2 and Supplementary Files S10, S11. Four major clusters were formed by transcripts with increased expression (Supplementary Figure S2A) and GO terms in these clusters were associated with ‘metabolism and gene expression’, ‘intracellular/nuclear localization’, ‘chromosome/chromatin’, and ‘macromolecular metabolism’. The downregulated transcripts of segment 3 were related to ‘signaling in immune system’, ‘cell-mediated and innate immunity’, ‘vesicle, vacuole and ER’, and ‘ECM’ (Supplementary Figure S2B). Segment 4 (Figure 2) representing exclusive DEGs of ATT versus PRE mainly featured a large cluster possessing terms of fundamental cellular processes associated with ‘chromosome and cell cycle’ for upregulated transcripts (Supplementary Figure S3A; Supplementary File S12), and only a platelet-related GO cluster for downregulated transcripts (Supplementary Figure S3B; Supplementary File S13).

**Figure 6:**
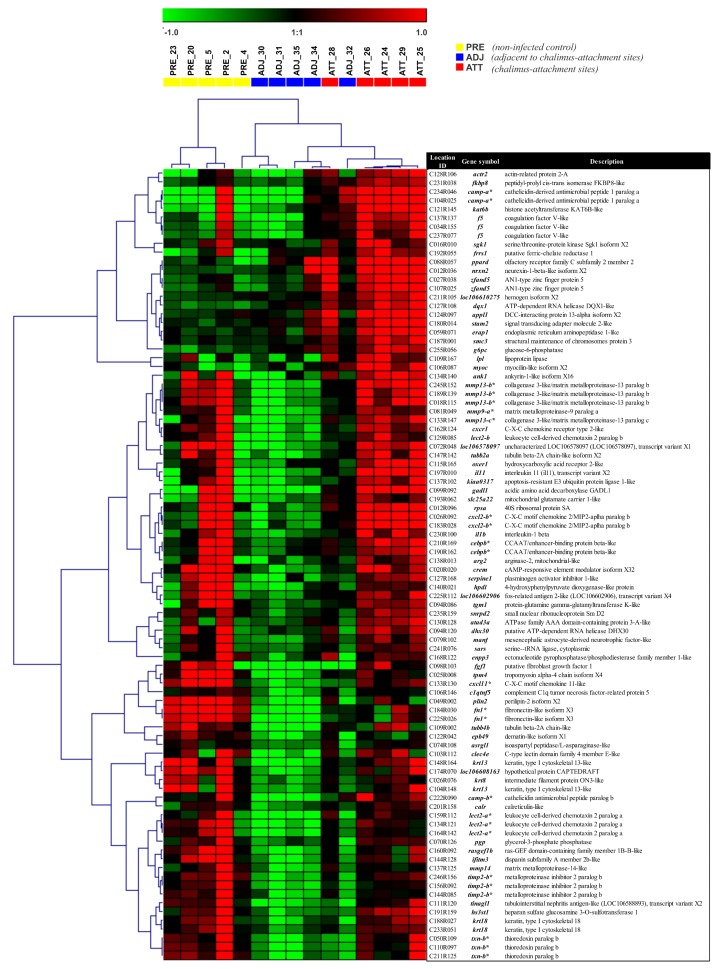
Heatmap illustration and hierarchical clustering analyses of DEPs between ATT and ADJ sites in Atlantic salmon fins across treatment groups. Clustering and heatmap results as an illustration of high-resolution figures are available in Supplementary Figure S6. Rows and columns represent the log_2_ fold-change values of different transcript expression levels (90 DEPs) and individual fish from the lice-infection groups (colored boxes), respectively. Genes were median-centered and clustered using Pearson correlation and complete linkage hierarchical clustering. The colored boxes below the top legend represent individual fish from the lice-infection groups. *, transcripts that were QPCR-assayed.

Enrichment analyses performed on DEGs with FC ≥ |2| from the entire chalimus-responsive DEPs (*n* = 1014) with ClueGO are available in Supplementary Figure S4A and Supplementary File S14. ‘Regulation of immune system process’ (GO:0002682) was the hub for 3 clusters: ‘cell-mediated immunity and defense’, ‘cell migration and adhesion’, and ‘development and morphogenesis’. Other enriched clusters in this pathway network included ‘ECM/collagen degradation’, ‘metabolism’, ‘extracellular’, ‘ER’, ‘complement system’, and ‘signaling’. A total of 163 DEPs contributing to the hub GO term, ‘regulation of immune system process’ (GO:0002682), were used in constructing a heatmap (Supplementary Figure S4B) that revealed the transcript profile of many immune-relevant transcripts (110 DEGs). An overview of GO/pathway term networks analyses demonstrated an overall pattern of immune suppression at both ADJ and ATT sites (by means of downregulating major defense-relevant GO pathways compared to PRE; Figure 4B and Supplementary Figure S2B) and induction of anti-lice responses at ATT sites (by means of upregulating some immune-related GO pathways compared to ADJ; Figure 5 and Figure 6).

### 2.5. Identification of Dysregulated Pathways

The GO/pathway term network analysis identified 249 enriched GO terms. We were able to annotate over half (*n* = 134) of these enriched GO/pathway terms based on the C5 collection of Molecular Signatures Database (MSigDB; e.g., ‘defense response’, ‘metalloendopeptidase activity’, and ‘innate immune response’). These 134 enriched pathways and the expression data of contributing DEGs were analyzed using the Pathifier package [34] to calculate the Pathway Deregulation Score (PDS) for each sample, and samples were clustered with pathways (Supplementary Figure S5A). With respect to PRE control, the degree of dysregulation was demonstrated to be different among ATT and ADJ fin sites; however, the latter two groups were found to have no distinct separation. It was evident that the ADJ fin sites show a higher degree of dysregulation when compared with ATT fin sites (*p* < 0.05), at least in part, based on the available PDS of 134 GO/pathways, including many immune-relevant GO terms, such as, ‘innate immune response’ (GO:0045087), ‘response to wounding’ (GO:0009611), and ‘chemokine-mediated signaling pathway’ (GO:0070098) (Supplementary Figure S5B–J).

### 2.6. Chalimus-Responsive, Differentially Expressed Transcripts among Different Fin Sample Groups

Heatmaps and clustering analyses provided further visual insights into individual DEPs/DEGs, and their overall and specific expression patterns across sample types. Different DEP sets were subjected to cluster analyses and heatmaps were generated to evaluate if the sample types could be distinguished based on the transcriptional expression profiles (Figure 6 and Supplementary Figure S6). Direct comparison of ATT and ADJ (90 DEPs) showed that many immune-relevant transcripts were significantly upregulated in ATT sites (e.g., several matrix metalloproteinases (*mmp*s), and their inhibitory counterpart (*timp2*), cathelicidin (*camp*), leukocyte cell-derived chemotaxin-2 (*lect2*), C-X-C chemokine 2 (*cxcl2-b*), and interleukin 1-beta (*il1b*); Figure 6) compared with ADJ.

Selected transcripts with important biological roles (based on the enriched functional classes) that demonstrated distinct transcription in terms of pattern and/or magnitude are shown under different functional categories in Table 1 and Table 2. Transcriptionally modulated genes were mainly associated with gene expression (transcription and translation), metabolic processes (biosynthesis and catabolism of macromolecules), melanin biosynthesis, ECM organization and disassembly, oxygen transport, redox homeostasis (Table 1), and several components of the immune system (Table 2). The majority of the selected DEGs were present in segments 1 and 2 (Figure 2).

Transcription of a large number of homeobox protein family members (*hox*) was affected, where some of them were upregulated (e.g., *hoxa5*, *hoxc9*) while others were downregulated (e.g., *hoxa9*, *hoxc11*, *hoxd12*) in both ADJ and ATT sites compared to PRE (Table 1). CCAAT/enhancer-binding protein beta (*cebpb*) was induced in ATT sites compared to ADJ sites. Two other transcriptional regulators (i.e., zinc finger protein gfi-1b, *gfi1*, and peroxisome proliferator-activated receptor delta, *ppard*) were among the most-induced transcripts in chalimus-attachment sites compared to PRE (Table 1). Sugar or energy metabolism was one of the many metabolic pathways affected during infection, and key enzymes associated with energy metabolism appeared to be upregulated in ATT and ADJ compared to PRE including transketolase (*tkt*), glucose-6-phosphatase (*g6pc*), fructose-bisphosphate aldolase C (*aldoc*), NADH dehydrogenase subunit 1 (*nd1*), and methyltransferase-like protein 17 (*mettl17*) (Table 1). We also found that members of the lipoxygenases (LOX; e.g., *aloxe3* (Table 1); *alox12*, *alox15b* (Supplementary File S1)) family were upregulated in ADJ and ATT sites compared to PRE control. Three key enzymes involved in melanin biosynthesis (i.e., *tyrp1*, *dct*, *tyr*) were markedly downregulated in both ADJ and ATT sites compared to PRE (Table 1).

Substantial changes in the expression of genes encoding structural components of the cytoskeleton and ECM constituents were also observed (Table 1; Supplementary File S1). Genes encoding collagens (e.g., *col10a1*, *col6a1*, *col15a1*), actins (e.g., *tpm1*), tubulins (e.g., *tuba4a*, *tubb*), fibronectin (*fn1*), biglycan (*bgn*), and decorin (*dcn*) were mostly downregulated in both ATT and ADJ compared to the PRE control, whereas some laminin subunit genes were upregulated in ADJ compared to PRE (*lamb1*, *lamb4*) (Table 1; Supplementary File S1). We observed decreased transcription of regulators of matrix remodeling (*tfpi2*; Supplementary File S1), and enzymes degrading structural components of ECM (e.g., *mmp*s: *2*, *9*, *13*, *19*; serine proteases: *htra1*, *htra3* and cathepsins: *cts*) in ATT and ADJ compared to PRE (segment 1; Table 1). However, it should be noted that both *mmp*s (*mmp9-a*, *mmp13-b*, *mmp13-c*, *mmp14*) and *timp2-b* demonstrated an induced expression in ATT compared to ADJ (segment 2). Some genes associated with oxygen transport (hemoglobin subunits: *hba*, *hba4*, *hbb*) and iron metabolism (5-aminolevulinate synthase, *alas2*) also presented reduced transcript levels in lice-infected salmon (ATT and ADJ) compared to PRE. However, an iron-binding protein of this group, cytoglobin-2 (*cygb*), was upregulated in ADJ sites compared with PRE (segment 3; Table 1). Several members of the antioxidant enzyme family were found to be affected during lice infection. The transcription of thioredoxin-related (*txn-b*, *txndc5*), peroxiredoxin (*prxl2a*), glutathione peroxidase (*gpx7*), glutathione S-transferase (*gsta*), and ferritin (*fth1*, *frim*) genes was decreased in ADJ sites compared to PRE. Although the transcription of many of these genes in ATT compared with PRE was also reduced (segment 1; *txndc5*, *prxl2a*, *gpx7*, *gsta*), expression of *txn-b* was higher in the ATT site compared to the ADJ site (segment 2; Table 1).

In the present work, dramatic changes in various immune-relevant pathways and mechanisms were seen post-lice exposure (Table 2). Decreased mRNA abundance of various pattern recognition receptors (PRRs) was observed (different cluster differentiation antigens, *cd93*, *cd209*, *cd302*; galectin-1, *lgals1*; proteins involved in mannose recognition, *mbl2,* and *mrc1*; two C-type lectin members, *clec4m* and *clec4e*). Chalimus-attachment induced the expression of *lect2*, C-X-C chemokines (*cxcl2*, *cxcl11*), receptors (e.g., *cxcr1*), and interleukins (*il1b* and *il11*) in ATT compared to ADJ. A C-C chemokine (*ccl4*) and a receptor (*cmklr1*) were downregulated during infection (both in ATT and ADJ) compared to PRE. Genes encoding different complement factors (cf such as *cfh*, *c4a*, *cfd*, but not *cfb*) also showed reduced expression in ATT and ADJ compared to PRE (segment 1; Table 2). Further, downregulation was noticed for complement receptor, *c3ar1*, along with *cd59*, in ATT and ADJ compared to PRE. Players in the coagulation cascade demonstrated a differential response to sea lice. Coagulation factor X (*f10*) was induced; however, *f3* was downregulated in ATT and ADJ compared to PRE (segment 1; Table 1). Meanwhile, increased transcription of *f5* and plasminogen activator inhibitor 1 (*serpine1*) at ATT sites was observed compared to ADJ (Table 1). A panel of mRNAs encoding proteins involved in inflammation and APR (e.g., vascular cell adhesion protein 1, *vcam1*; midkine, *mdk*; C-reactive protein, *crp*; prostaglandin E synthase, *ptges*) was found to be downregulated in ATT and ADJ compared to PRE. However, the gene encoding prostaglandin D2 receptor 2 (*gpr44*) was upregulated in ADJ versus PRE fin samples (Table 2). The downregulation of viral-responsive genes (e.g., *rsad2-a* (alias viperin); and two interferon-induced proteins, *ifi44* and *ifit5*, transcripts encoding TRIM family members, *trim8* and *trim58*) in both ATT and ADJ compared to PRE, was a characteristic response to chalimus infection. Nevertheless, interferon α3 (*ifna-a*) mRNA increased in ATT and ADJ compared to PRE (Table 2). A set of innate immune genes demonstrated significantly reduced transcription either in the ADJ site alone (complement component 1 Q binding protein, *c1qbp* and *camp-b*) or both in ADJ and ATT sites (putative defense protein HDD11, *hdd11*; CD99 antigen-like protein 2, *cd99*; complement c1q tumor necrosis factor-related protein 6, *c1qtnf6*; fibrinogen-like protein 1, *fgl1*; lipopolysaccharide-binding protein, *lbp*; lipopolysaccharide-induced tumor necrosis factor-alpha, *litaf*) compared to PRE (Table 2). In contrast, NLR family CARD domain-containing protein 3 (*nlrc3*) and complement C1q tumor necrosis factor-related protein 3 (*c1qtnf3*) showed induced expression in ATT and ADJ compared to PRE. Two *camp* paralogs, fibroblast growth factor 1 (*fgf1*) and arginase-2 (*arg2*) genes, demonstrated significantly higher expression in ATT to that of ADJ. Genes encoding important components of adaptive immunity, such as two major histocompatibility (MH) class I antigens (*hlab* and *hlah*) and an immunoglobulin chain (e.g., *iglc3*), were also significantly downregulated in ATT and ADJ compared to PRE.

### 2.7. QPCR Confirmation of Selected Transcripts

**Figure 7:**
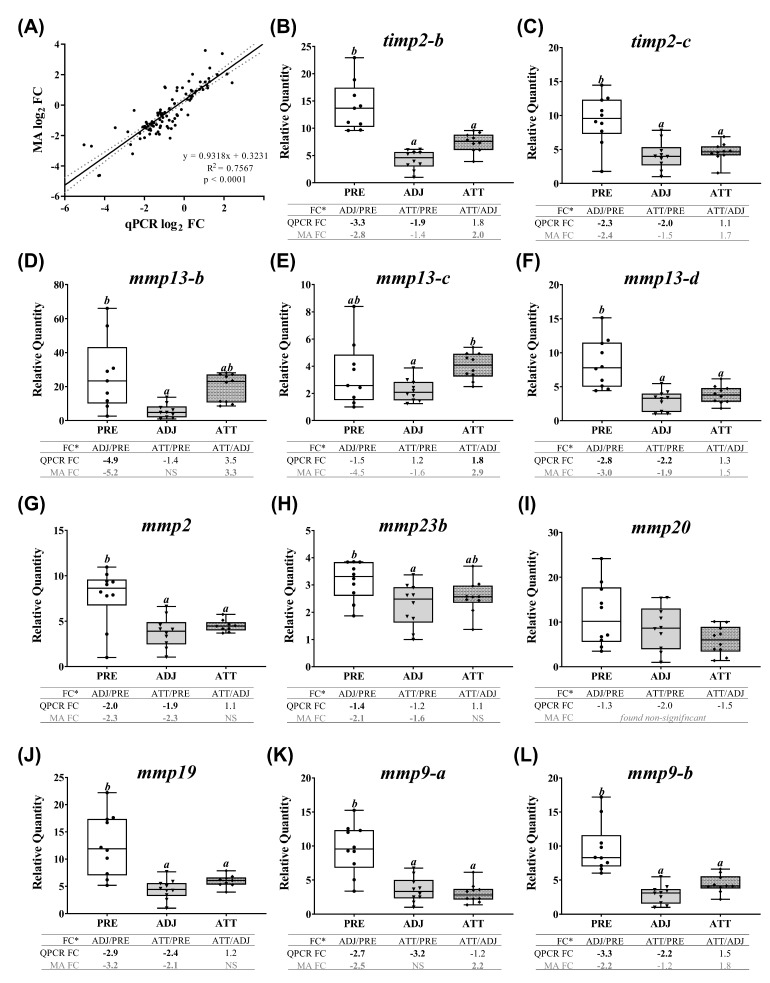

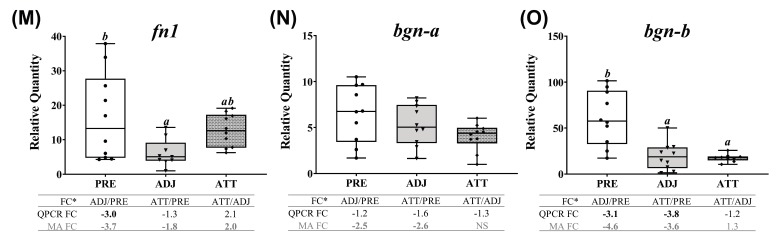
Correlation between the microarray and real-time quantitative polymerase chain reaction (QPCR) data, and QPCR confirmation of selected microarray-identified genes in theme 1 (Table 1 and Table 3) associated with ECM, tissue repair, and wound healing. (**A**) Scatterplot of log_2_-transformed gene expression fold-changes (FC) between treatment groups calculated from the microarray log_2_ ratios and log_2_-transformed QPCR relative quantity (RQ) ratios for all the QPCR-assayed transcripts. Each dot represents either an ADJ versus PRE, ATT versus PRE, or ATT versus ADJ comparison (i.e., one biological replicate) for a given target transcript. (**B**–**O**) Boxplots of QPCR data for the abundance of selected transcripts associated with ECM degradation, tissue repair/remodeling, and wound healing. Plots reveal median RQ values and interquartile ranges. Different letters above bars represent significant differences between groups (one-way analysis of variance (ANOVA), Tukey’s post hoc test, *p* < 0.05). Inset table below each plot shows FC of GOI from microarray and QPCR. Numbers with negative sign represent fold downregulation calculated as the inverse of FC (i.e., −1/FC) for the values less than one. When more than one probe represents a transcript in the microarray, the average FC is shown in the inset table. Numbers of probes contributing to the average FC are provided in Table 1 and Table 2. Bold letters indicate that a FC is statistically significant in QPCR or microarray by ANOVA and modified *t*-test, respectively. NS, found non-significant in the microarray. Refer to the heatmaps for each theme provided in Supplementary Figure S7. MA, microarray.

**Figure 8:**
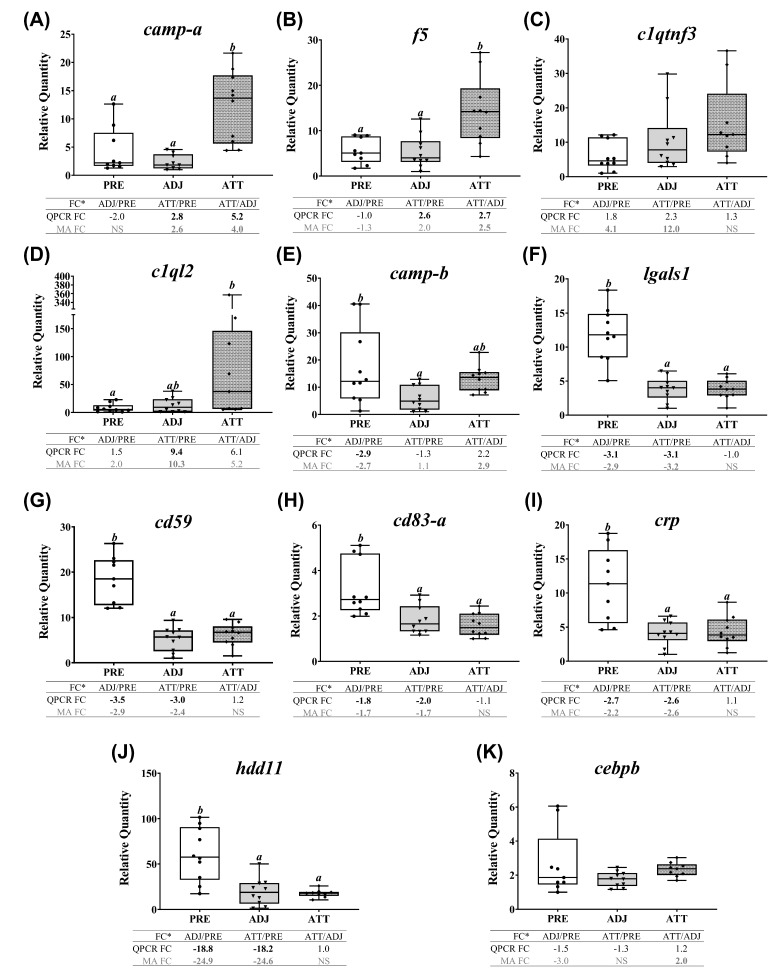
QPCR confirmation of selected microarray-identified transcripts in theme 2 (Table 2 and Table 3) that are primarily associated with immunity and defense (not including antiviral response). For details of the captions, refer to Figure 7.

**Figure 9:**
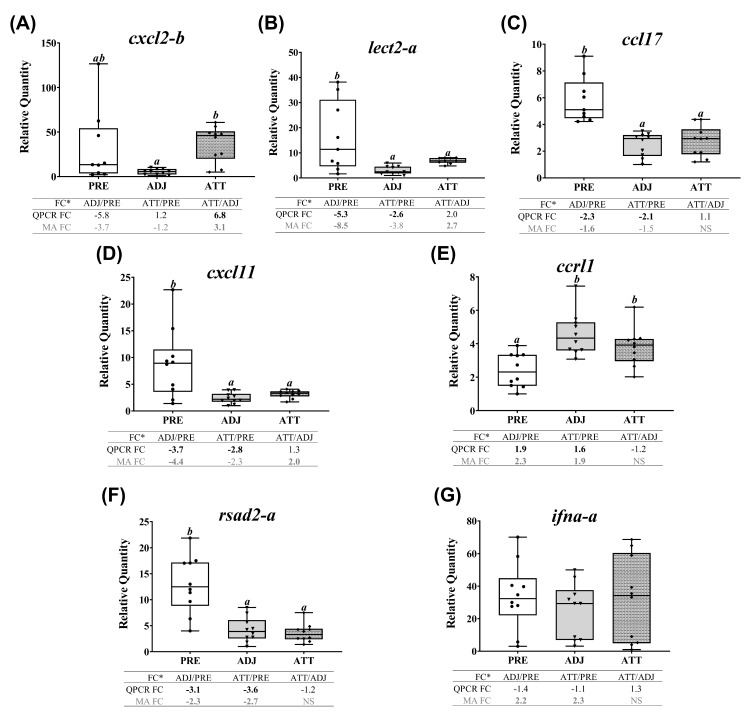
QPCR confirmation of selected microarray-identified transcripts in themes 3 and 4 (Table 2 and Table 3). Transcript abundance of selected transcripts associated with chemotaxis and signaling (theme 3), and antiviral response (theme 4). For details of the captions, please refer to Figure 7, (*cxcl2 (alias *mip2*, macrophage inflammatory protein 2) also demonstrates homology with *cxcl8*/interleukin 8 (*il8*)).

**Figure 10:**
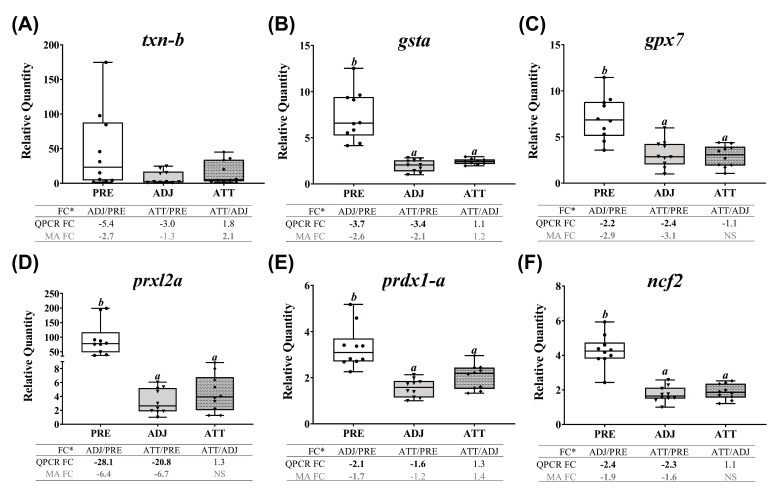
QPCR confirmation of selected microarray-identified transcripts in theme 5 (Table 1 and Table 3) that are associated with antioxidant activity and redox homeostasis. For details of the captions, refer to Figure 7.

A subset of chalimus-responsive transcripts (37 microarray-identified and one additional MMP family member) was chosen for confirming the microarray results by QPCR. These transcripts were associated with five different functional themes including (1) ECM degradation, tissue repair/remodeling, and wound healing, (2) immunity and defense, (3) chemotaxis and signaling, (4) antiviral response, and (5) antioxidant activity and redox homeostasis (Table 3). In addition to the samples used in microarray, we included 4 more samples/group in the QPCR analysis (*n* = 10 in total/group). Log_2_-transformed microarray and QPCR FC showed a significant positive correlation (R^2^ = 0.76, *p* < 0.0001; Figure 7A). Individual relative quantity (RQ) profiles for QPCR with FC values from both QPCR and microarray for each theme are presented in Figure 7, Figure 8, Figure 9 and Figure 10. Based on inset tables, the FCs obtained from two techniques presented some divergence across genes of interest (GOIs), but very similar patterns in the direction of change (i.e., up- or down-regulation), in general (Figure 7, Figure 8, Figure 9 and Figure 10). Specifically, 32 transcripts (86.5%, except *bgn-a*, *c1qtnf3*, *cebpb*, *ifna-a*, and *txn-b*) were confirmed as significantly (*p* < 0.05) differentially expressed in at least one comparison among three sample groups (i.e., ADJ versus PRE, ATT versus PRE, or ATT versus ADJ), from which they were identified as informative DEPs by SAM (Supplementary File S1).

QPCR profiles of transcripts involved in ECM degradation, wound healing, and tissue repair/remodeling (theme 1) are shown in Figure 7. The majority of these transcripts demonstrated lower expression in infected animals compared to PRE control animals. Significant differences (*p* < 0.05) were observed in both ATT and ADJ fins for *timp2-b*, *timp2-c*, *mmp13-d*, *mmp2*, *mmp19*, *mmp9*-a, *mmp9*-b, and *bgn-b*. Except for *mmp9-a*, *mmp20*, and *bgn* paralogs, the expression of other transcripts in ATT was higher compared to ADJ, although this was only significant (*p* < 0.05) for *mmp13-c* based on QPCR results (Figure 7E). It was evident that, in general, transcripts in theme 1 were expressed at a lower level in ADJ compared to PRE (and ATT for some transcripts) (Supplementary Figure S7A).

Conversely, the transcripts of theme 2 that are immune/defense (not including antiviral response)-relevant demonstrated two distinct patterns of expression (Figure 8), as revealed by a heatmap (Supplementary Figure S7B). A subset of transcripts (i.e., *camp-a*, *f5*, and *c1ql2*) were more highly expressed in ATT compared to ADJ and PRE. Significant differences (*p* < 0.05) in mRNA levels between ATT and ADJ were only detected for *camp-a* and *f5* by both QPCR and microarray (Figure 8A,B). Although it showed a similar trend with the microarray data, *clqtnf3* transcription did not differ significantly among sample groups (Figure 8C). The mRNA level of *c1ql2* was significantly higher in ATT compared with PRE (*p* < 0.05; FC > 9; Figure 8D). The second set, comprising *camp-b*, *lgals1*, *cd59*, *cd83*, *crp*, and *hdd11* transcripts, showed significant (*p* < 0.05) lower expression in ADJ and/or ATT compared with PRE (Figure 8E–J). QPCR failed to detect any significant difference in the expression of *cebpb* (Figure 8K).

Many transcripts of both themes 3 (chemotaxis and signaling) and 4 (antiviral response) showed similarly repressed expression in ADJ and ATT, as of theme 1, when compared with PRE samples, except for *cxcl2-b*, *ccrl1* and *ifna-a* (Figure 9, Supplementary Figure S7C). Chalimus-attachment significantly (*p* < 0.05) upregulated the transcript level of *cxcl2-b* in ATT compared to ADJ (Figure 9A). While the expression level of *lect2-a*, *ccl17*, *cxcl11* and *rsad2-a* was significantly (*p* < 0.05) downregulated in ADJ and ATT, compared with PRE (Figure 9B-D,F), *ccrl1* was significantly (*p* < 0.05) upregulated (Figure 9E) in both ATT and ADJ compared to PRE controls. According to QPCR data, *ifna-a* was transcriptionally non-responsive to chalimus infection (Figure 9G).

Finally, QPCR (and microarray) results showed that chalimus infection robustly and significantly (*p* < 0.05, except *txn-b*, which showed non-significant QPCR results) suppressed the expression of all the studied transcripts representing antioxidant and redox system in theme 5 (Figure 10). Both QPCR and microarray did not find significant (*p* < 0.05) differences between ATT and ADJ sites in fins for *gsta*, *gpx7*, *prxl2a*, *prdx1-a*, and *ncf2*. However, as illustrated in the heatmap (Supplementary Figure S7D), transcription of theme 5 genes appeared to be more highly suppressed in ADJ compared to that of ATT (with respect to PRE; see FCs).

### 2.8. Multivariate and Correlation Analyses Based on Gene Expression and Phenotype Data

**Figure 11:**
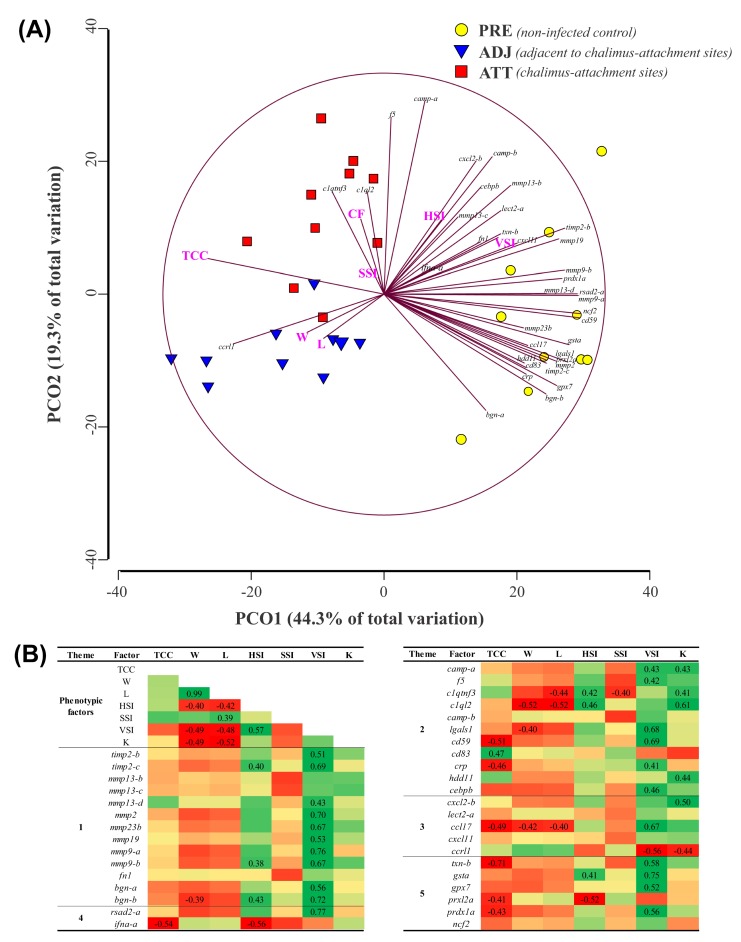
Principal coordinate analysis (PCoA) and correlation analysis of fin transcript expression data from QPCR with phenotypic factors. Standardized and log_2_-transformed data, respectively, were used in PCoA and correlation analyses. (**A**) Similarities and dissimilarities among treatment groups revealed by PCoA performed by PRIMER 6.1.15. (**B**) log_2_-transformed data were subjected to factor analysis using IBM SPSS Statistics (v25). Matrix represents the Pearson correlation coefficients between 8 phenotypic factors (columns; e.g., chalimus count) and both fin gene expression and the phenotypic factors (rows). Positive and negative correlations are indicated with green and red, respectively. A number for the corresponding correlation is displayed if the correlation is significant (*p* < 0.05). TCC, total chalimus count; W, weight; L, length; HSI, hepatosomatic index (HSI = 100×(liver weight/W)); SSI, spleen-somatic index (SSI = 1000× (spleen weight/W)); VSI, viscerosomatic index (VSI = 100× (viscera weight/W)), and K, Fulton’s condition factor (K = 100× (W/L^3^)).

To examine the similarities and dissimilarities among the fin sample groups, and to understand the relationship among different phenotypic parameters, we performed a PCoA. As shown in Figure 11A, PCoA explained 63.6% of the variation among sample types. While PCO1, driven by many transcripts (e.g., *mmp9-a*, *mmp13-d*, *cd59*, *ncf2*, *rsad2*, and *prdx1a*) segregated PRE control salmon fins from infected salmon fins, PCO2 was greatly influenced by *f5* and *camp-a*, and separated fin samples of infected animals based on sampling sites (i.e., ATT versus ADJ). Permutational multivariate analysis of variance (PERMANOVA) indicated that the sample groups are significantly different from each other (*p* = 0.0001). Higher expression of the majority of the QPCR-assayed genes in the PRE group was also evident from PCoA. To complement PCoA, we conducted Pearson correlation analyses. Seven different phenotypic parameters and gene expression data were used in this correlation analysis, and the results are tabulated in Figure 11B. Chalimus load negatively correlated with many genes that were QPCR-tested (e.g., *ifna-a*, *cd59*, *crp*, *ccl17*, *txn-b*, *prxl2a*, and *prdx1a*). Conversely, *cd83* revealed a positive correlation with the chalimus load. Interestingly, viscerosomatic index (VSI) was positively and significantly (*p* < 0.05) correlated with 22 genes tested, while negatively and significantly (*p* < 0.05) correlated with *ccrl1* (Figure 11B).

## 3. Discussion

Anti-parasitic responses in teleosts have received less attention compared to studies focused on responses to bacterial and viral infections [35]. There is little published information available on early immune responses of Atlantic salmon against sea lice (*L. salmonis*) [24,25]. We used a moderate-density infection model (ca. 0.15 *L. salmonis*/g fish) in our study. Johnson and Albright [36] reported an abundant presence of early life stages (<5 dpi) of *L. salmonis* on gills of Atlantic salmon, while later life stages (10–20 dpi) preferentially settled on fins when compared to gills and other body surfaces. A comparative study reported that *L. salmonis* and *C. elongatus* chalimi were abundantly attached to fins compared to body and head, with the lowest abundance in gills under natural conditions in Atlantic salmon, whereas gills were a major attachment location in experimental models compared to fins and body [37]. Chalimi distribution in our infection trial was in agreement with these studies [36,37], and a considerable number of chalimi settling on the gills could be an artifact of laboratory conditions [37]. By being the initial and preferential settlement sites of chalimi, fins were targeted for gene expression profiling in the present study.

Some studies have examined the spectrum of relative susceptibility in different salmonid species to *L. salmonis* infection. Results of these studies highlighted that some salmonids (e.g., chinook (*O. tshawytscha*), chum (*O. keta*) or sockeye (*O. nerka*) salmon) in addition to the Atlantic salmon are highly susceptible, whereas others are more resistant (e.g., coho and pink salmon) [8,9,36,38,39,40]. The high infection rate and prolonged retention of parasites throughout the trial period in our study (i.e., chalimi versus pre-adults counts) confirmed Atlantic salmon’s inability to reject sea lice and its status as a susceptible host [2].

**Figure 12:**
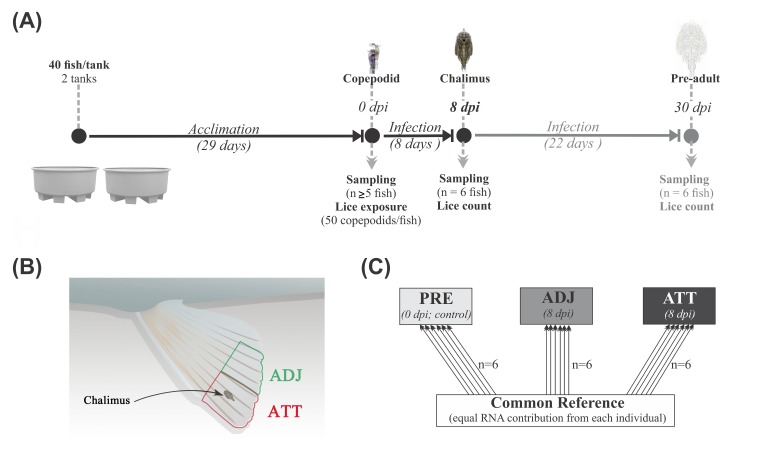
Overview of infection trial, sampling, and microarray design. (**A**) The design of the sea lice infection experiment. Sampling points and corresponding developmental stages of lice are indicated along the timeline (dpi, days post-infection). (**B**) Schematic sketch of fin sampling (not drawn to scale). Pelvic fin tips were sampled from chalimus-attachment (ATT) and adjacent (ADJ) sites. (**C**) Common reference-based microarray experimental design. Arrows represent the biological replicates used from three treatment groups (PRE, fins from control fish prior to lice exposure (*n* = 6); ATT and ADJ, fins from chalimus-attachment and adjacent sites, respectively, both sampled from same fish (*n* = 6)). The base and arrowhead show the Cy3-labelled reference pool and Cy5-labelled experimental samples, respectively.

It is difficult to determine the gross changes in whole tissue transcriptome in response to parasites using the conventional targeted QPCR approach. However, microarray-based high-throughput platforms are used to screen tens of thousands of genes and hundreds of pathways simultaneously and to identify and characterize known and putative markers. Herein, we focused on 8 dpi (Figure 12A), at which time copepodids had developed to the chalimus I stage. We employed microarray techniques to identify the differentially expressed transcripts and elucidate the pathways and mechanisms involved in early response against sea lice attachment on Atlantic salmon fins.

The results of two independent approaches (microarray and QPCR) were generally in significant concordance. However, we observed some transcripts that were differentially expressed as per SAM (e.g., *bgn-a*, *c1qtnf3*, *cebpb*, *ifna-a*, and *txn-b*) but with no significant differences in RQs from QPCR across treatment groups, or vice versa (Figure 7, Figure 8, Figure 9 and Figure 10). This divergence may have been caused by different statistical approaches used in microarray (i.e., SAM (a modified *t*-test) for pairwise comparison of treatment groups) and QPCR (i.e., analysis of variance (ANOVA) compared the means of three independent groups). The increased number of biological replicates enrolled in the QPCR study could also be a source of this disagreement.

Transcriptional differences between paralogs of *bgn*, *camp*, *mmp9*, *mmp13*, and *timp2* were addressed in the current study. Paralogs of some genes demonstrated similar mRNA expression profiles (e.g., *timp2*, *mmp13* (paralogs b and d), and *bgn*), whereas others presented distinct transcript expression profiles (e.g., *mmp9*, *camp*) (Figure 7, Figure 8, Figure 9 and Figure 10). The entire teleost lineage has undergone three rounds (1R, 2R, 3R) of whole-genome duplication (WGD) that shaped their evolution. While 3R was a teleost-specific WGD, a salmonid-specific fourth WGD (4R) occurred in the common ancestor from which different sub-lineages of salmonids evolved [41,42]. These WGDs have generated a complex and diverse repertoire of paralogs from ancestral genes in salmonids. The potential fates of duplicated genes post-WGD include subfunctionalization (each paralog retaining a subset of its original ancestral function) or neofunctionalization (a paralog acquiring a new function) [41,42]. The paralog-specific transcript expression patterns from our study provide evidence for gene duplication and divergence. However, whether these paralogs were subfunctionlized or neofunctionalized is not known and requires functional studies with the encoded proteins.

Previous research found that local response in fin/skin to lice was more diverse and complex when compared with the systemic response (such as in spleen) [24]. In the current study, 6568 DEPs were identified (Figure 1B; representing 3571 DEGs), and the larger fraction of DEPs was present in ADJ versus PRE compared to ATT versus PRE and ATT versus ADJ. Quantitative estimation of pathway dysregulation indicated that the ADJ group presents higher PDS compared to ATT and PRE groups for the majority of the GO terms, including many immune-relevant GO terms (Supplementary Figure S5), and this is in agreement with the relative distribution of DEPs among sample groups (Figure 1B), PCoA of DEPs (Figure 1C), and the RQ values of the majority of GOIs determined by QPCR (Figure 7, Figure 8, Figure 9 and Figure 10). Several aspects of gene expression profiles observed in our study provided evidence suggesting that specific immune signaling pathways were modulated in infected fins (to reject sea lice). It was interesting to note that ~30% of the entire set of chalimus-responsive DEPs (1928, segment 1; Figure 2) were common among ATT and ADJ compared to PRE, and each of them varied only in the level of mRNA expression, not in their direction of modulation (Supplementary File S1).

Our expression profiling and enriched GO/pathway term analyses collectively uncovered that chalimus infection caused transcriptional changes in an array of biological processes (Table 1 and Table 2), some of which are discussed below.

### 3.1. Chalimus Infection Influences Transcription Machinery, Energy Metabolism, and Melanin Biosynthesis

As noticed in our GO enrichment analyses (*gene expression* (GO:0010467); Supplementary File S7), a wide range of homeobox transcription factors (TFs) has been seen to be modulated by stress conditions, including infection by sea lice in salmon [25,26]. Although members of the HOX family were originally found to determine cell fate and regulate organ development [43], recent discoveries have implicated them in wound healing and tissue remodeling [44]. CEBP is a family of TFs governing immune and inflammatory responses [45], and *cebpb* (encoding CEBPβ protein) was proposed as a lice-responsive marker in salmonid skin by many studies [23,39]. This transcript was upregulated in skin of Atlantic salmon as early as 24 hpi with adult *L. salmonis* [38]. NF-κB is another vital synergetic TF of CEBPβ that activates inflammatory responses, and *nfkb1* and *nfkb2* mRNAs were significantly upregulated in ADJ sites compared to PRE (~1.5-fold; Supplementary File S1). The upregulation of *gfi1b* in ATT and ADJ compared to PRE may lead to transcriptional repression in erythroid cells, as shown in mouse [46]. PPARD is a member of the ligand-inducible PPAR TF family whose increased transcription might enhance fatty acid oxidation and energy uncoupling in muscle and adipose tissues, and suppress macrophage-derived inflammation [47,48]. Modulated expression of transcripts encoding all these TFs, which are involved in a multitude of functions, such as cytokine production, inflammation, wound healing, and hematopoiesis, was suggestive of major changes in multiple downstream events in our study.

Based on our GO/pathway term analyses, enriched GO term clusters represented by the upregulated DEGs in the ATT and ADJ compared to PRE (e.g., ‘metabolism’, ‘cell cycle and chromosomes’, and ‘cytoskeleton’) implied that fin regeneration processes might be in progress in lice-infected fins (Figure 4A, Supplementary File S7). Gross changes in metabolic processes of various macromolecules (sugars, amino acids, and nucleic acids) were apparent, particularly in ADJ and ATT sites compared to PRE (Group 3; e.g., heterocycle metabolic process (GO:0046483), Supplementary File S7). Induced transcription of genes involved in sugar and lipid metabolism in infected animals compared to PRE suggested an increased energy mobilization during infection. Energy metabolism-related genes were also present among DEGs in *Caligus*-infected salmon fins [25]. Upregulated expression of several transcripts encoding LOX enzymes (particularly, *aloxe3* and *alox12*; Supplementary File S1) that play key roles in biosynthesis of oxylipin signaling molecules [49] suggested that the salmon attempted to modulate the permeability barrier function of fin against sea lice.

Our enrichment analyses indicated that melanin-related pathways are downregulated in lice-infected salmon (e.g., *melanosome* (GO:0042470); Figure 4B, Supplementary File S8). Kittilsen et al. [50] showed that hyper-melanized salmonids demonstrate a reduced physiological and behavioral response to stress. In addition, the same group later reported that melanin-based pigmentation significantly correlated with the immunocompetence of Atlantic salmon and its resistance to sea lice [51]. A recent study found hyperpigmentation and migrating pigment bodies at the wound surface and implicated it with wound repairing processes in Atlantic salmon [52]. It may be argued that parasite-mediated inhibition of melanin synthesis could be a potential mechanism supporting the lice pathology.

### 3.2. Sea Lice Modulate ECM Integrity and Catabolism, Wound Repair, Inflammation, Acute Phase Response, and Coagulation

Complex dynamics in transcriptional expression of different structural and functional components of ECM were evident from our GO/pathway term and expression analyses (e.g., group 5 representing ECM; Supplementary File S8). Notably, transcripts encoding several structural elements of ECM, such as fibronectin, collagen chains, laminin, and proteoglycans (BGN, DCN), were downregulated, suggesting impaired tissue repair and wound healing cascades at the infection site. Beyond its structural roles in ECM, BGN orchestrates signaling networks involved in inflammation and immunity [53]. Meanwhile, DCN is a multifunctional protein involved in anti-fibrosis by interacting mainly with TGFβ and other matrix molecules and cytokines (Reference [54] and references therein). Both BGN and DCN promote cell migration and are involved in the remodeling process [55]. Downregulation of *fn1* (fibronectin) in liver throughout the lice-infection led Skugor et al. [23] to suggest an impaired ability to heal wounds in salmon. Moreover, mRNAs encoding several lysosomal (serine (HTRA1, HTRA3) and cysteine (cathepsins)) proteases that are involved in ECM catabolism were also downregulated in ATT and ADJ compared to PRE. Annexins are important ECM proteins that play multiple roles in membrane scaffolding, cell division, signaling, and apoptosis [56], and genes of several annexin family members (e.g., *anxa13*) were downregulated in ATT and ADJ compared to PRE. As a collagen-specific chaperone, SERPINH1 plays roles in collagen biosynthesis and structural organization [57]. *serpinh1* mRNA demonstrated decreased abundance in ATT and ADJ compared to PRE.

The TGFβ proteins (a family of growth factors and cytokines), their receptor complexes, and associated proteins together build an intracellular transmission cascade that governs proliferation, differentiation, and migration of cells and determines the healing dynamics [58]. As noticed by Krasnov and colleagues [26], chalimus-attachment appeared to inhibit the TGFβ signaling network based on suppressed transcription of ligand (*tgfb3*), a receptor (*tgfbr2*), and several regulatory proteins (*lrrc32*, *tgfbi* [15], and *ltbp3*) of this signaling pathway (Supplementary File S1). Transcription of other growth factors and regulators that operate in conjunction with TGFβ, namely CTGF, FGF1 [59], and SERPINF1 [26], was also impacted in our study. Overall, these transcriptional responses may result in a lethargic wound repairing process.

There is an equilibrium in expression and activity between MMPs and their endogenous inhibitors (TIMPs) in the ECM microenvironment [60]. This balance primarily determines the dynamics of ECM degradation and remodeling. We identified a set of differentially transcribed *mmp*s (collagenases and gelatinases) along with two of their inhibitors (*timp2* and *timp3*). Paralog-specific QPCR assays were developed, and results indicated distinct expression patterns (Table 1, Figure 7). Although only QPCR confirmed for *mmp13-c*, the microarray results suggested a more pronounced expression of several *mmp*s (e.g., *mmp9-a* and *mmp13-b*) and *timp2-b* in ATT compared to ADJ. Previous evidence from different groups suggests that lice-infection chronically damages the tissues, resulting in a hallmark aberrant expression of *mmp*s [8,23,38,39]. Two enriched GO terms related to wound healing (i.e., response to wounding (GO:0009611) and wound healing (GO:0042060)) represented many associated genes that were downregulated in infected salmon compared with PRE (Figure 4B, Supplementary File S8). Collectively, these data suggest that the lice infection influences ECM catabolism, remodeling, and wound repair events in Atlantic salmon fin tissue.

Both microarray and QPCR indicated downregulated transcription of the gene encoding CRP, a well-known acute phase protein (APP) [61], in infected fin (Table 2, Figure 8I). Pro-inflammatory cytokines (e.g., IL1β, CXCL8) induce APR through the activation of NF-κB and CEBPβ [62]. Despite the upregulation of *il1b* mRNA, decreased expression of *crp* transcript in infected fin compared to PRE might have resulted in a suppressed APR during lice-infection. Limited local expression of *crp* in lice-attachment sites has been implicated with susceptibility of *S. salar* to sea lice based on comparison with resistant salmonids [24,38]. Decreased transcription of *mdk* implied an impaired tissue repair process as MDK enhances inflammation in mammals by promoting the migration of inflammatory leukocytes [63]. We found that transcription of *ptges* was also downregulated in ADJ and ATT compared to PRE (Table 2). Reduced transcription of genes encoding prostaglandin synthase enzymes (e.g., PTGES) in Atlantic salmon skin during lice-infection has previously been reported [24,38,39]. All these events may plausibly dysregulate inflammation by restricting and/or delaying it, which in turn may result in chronic wounds and higher susceptibility to parasitization [10,64]. This may lead to the cutaneous lesions and loss of osmotic balance in wounded areas, causing a greater amount of distress to infected salmon [65].

Some components of the coagulation cascade also play roles associated with APR (e.g., fibrinogen, plasminogen, and plasminogen activator inhibitor 1 (PAI-1)) [66] and inflammation (e.g., coagulation factor III and thrombin) [67]. As found in previous studies [24,68], members of the coagulation cascade, and hence hemostasis, were found to be affected by lice-infection in our study. We observed the downregulated transcription of *f3*, the primary initiator of the extrinsic coagulation pathway [69], and tissue factor pathway inhibitor 2 (*tfpi2*; [70]), in ATT and ADJ compared with PRE (Table 2). Although the blood-feeding characteristic of sea lice has been documented for matured developmental stages of sea lice [71], it is not clear whether or not the lice larvae possess any mechanisms to maintain a steady flow of host blood at feeding-sites. Conversely, a few genes encoding proteins with regulatory roles in the coagulation pathway, coagulation factor V (co-factor of factor Xa) and PAI-1 (that inhibits fibrinolysis), were induced in ATT compared to ADJ (Table 1). These findings suggest that hemostasis was dysregulated by chalimi, whereas salmon attempted to restore the coagulation mechanism at the chalimus-attachment site, perhaps to prevent the lice from blood-feeding.

### 3.3. Chalimus-Induced Changes in O_2_ Transport and Redox Homeostasis

The heme biosynthetic pathway is absent in many parasites [72]. Sea lice are hematophagous species that acquire iron from their hosts for their nutritional requirements. A ‘nutritional immunity’ concept hypothesized that hosts restrict the access of iron or heme groups by pathogens [73]. Evidence from different studies at the salmon–louse interface provided contrasting results. Heggland et al. [74] recently compared the gene expression of attached chalimi in gills and skin of Atlantic salmon and found elevated transcripts of genes important for absorption, storage, and/or transportation of iron and heme, digestive and detoxification enzymes, and anti-clotting elements in gills. Iron metabolism was shown to be modulated by means of increased transcriptional activities of genes associated with heme biosynthesis (e.g., *alas*) and iron transport (*fth1*, *frim*, haptoglobin (*hp*), and hepcidin (*hamp*)) coupled with decreased expression of enzymes degrading the heme (heme oxygenase, *hmox1*) [75]. However, other authors found coordinated suppression in systemic and/or local mRNA expression of hemoglobins, *alas2,* and *hmox1* [23,26]. In the current study, several probes representing hemoglobin subunits and *alas2* demonstrated decreased mRNA abundance post-infection compared to PRE (Table 1). Sustaining a lower blood flow and heme enrichment cannot be ruled out as a potential protective mechanism of the host to deprive the parasite of iron supply [26]. Additionally, proteins encoded by some of these genes (e.g., *hp*, *ferritin*, and *hamp*) are also considered to take part in APR [66]. The elevated transcript level of another oxygen-carrying protein, cytoglobin-2 (*cygb*), in ADJ compared to PRE (Table 1), suggested the existence of potential alternative mechanisms to protect the host tissues from hypoxia.

Excessive stress conditions, including pathogenic infections, could result in the production of a massive amount of reactive oxygen species (ROS). Although ROS play crucial roles at optimal physiological levels, they could cause damage to cells at higher concentrations [76]. Enzymatic antioxidants neutralize the excessive ROS and restore the cellular redox equilibrium. Many genes encoding antioxidant enzymes were transcriptionally suppressed (e.g., *gsta*, *gpx7*, *prxl2a*, and *prdx1-a*) by chalimi in infected animals compared to PRE, indicating that redox homeostasis was disturbed by the sea lice infection, as reported earlier [77]. However, in agreement with previous studies in salmonid skin [23,39], our microarray results revealed upregulated *txn-b* expression in ATT compared with ADJ (Supplementary Figure S4B). Collectively, these results suggest that TXN could be a crucial player in maintaining redox homeostasis during lice-infection.

### 3.4. Impact of Lice Infection on Expression of Immune-Relevant Transcripts

Although an overall downregulation of immune genes was a hallmark of lice pathology (Figure 4B, Supplementary Figure S4B; e.g., a large number of GO terms in groups 13 and 19 in Supplementary File S8), some immune markers were upregulated in ATT compared to ADJ (Figure 6; e.g., GO terms of groups 7 and 10 in Supplementary File S9).

#### 3.4.1. Pattern Recognition Receptors (PRRs)

PRRs play vital roles in innate immunity by recognizing pathogen- or damage-associated molecular patterns (PAMPs or DAMPs) [78]. These PRRs sense danger signals and initiate complex cascades culminating in the production of effector molecules, such as cytokines, ROS, antimicrobial peptides, growth factors, and complement proteins [79]. In line with previous studies [8,23,24,25,26], we found an overall transcriptional downregulation of several PRRs in infected salmon compared to PRE (e.g., *cd209*, *cd302*, *mbl2*, and *lgals1*). Expression of *mrc1* that encodes a mannose receptor was downregulated in ADJ compared with PRE (Table 2). As a member of the calcium-dependent C-type lectin receptor family (CLR) [80], CLEC4E is expressed in macrophages and dendritic cells [81]. The transcript encoding CLEC4E was downregulated in ADJ (compared with PRE) and upregulated in ATT compared with PRE. Two other members of CLRs, CD209 and CD302, are abundantly expressed in dendritic cells, and implicated with phagocytic pathogen-recognition [82] and dendritic cell migration [83], respectively. Mannose-binding lectins (e.g., MBL2 and MRC1) also belong to another subgroup of CTLs [84]. A recent study found a strong correlation between *mrc1* transcription and *C. rogercresseyi* chalimus-load in Atlantic salmon [25]. Galectin-1 (encoded by *lgals1*) is a glycan-binding protein that acts as a master regulator by controlling proinflammatory cytokine production, neutrophil trafficking, and eosinophil migration. Based on these findings, we speculate that lice-infected salmon could be vulnerable to secondary infections due to the compromised pathogen-sensing ability.

#### 3.4.2. Cytokine/Chemokine Signaling

The present study found altered transcriptional patterns for a large number of cytokines/chemokines and their receptors. A subset of these genes demonstrated downregulation in both ATT and ADJ compared to PRE (e.g., *ccl4*, *cmklr1*) (segment 1; e.g., *signal transduction* (GO:0007165), Supplementary File S8). CCL4 (MIP1β) is a potent lymphocyte chemoattractant, and activation of chemotaxis by CCL4 towards the injury site has been demonstrated in mammals [85,86]. Primarily expressed by dendritic cells and macrophages, CMKLR1 transduces the signals by chemerin, which is an antimicrobial molecule expressed in human skin and induces transmigration of various immune cells [87]. The CCR4–CCL17 receptor–ligand pair showed decreased expression in ADJ compared to PRE (segment 3; Figure 2), indicating coordinated changes in its signaling axis. In mammals, CCR4 is the receptor for CCL17 and is expressed in skin-homing distinct T cell subsets, including activated T cells, Th2 cells, and Treg cells, and implicated in skin-associated immune responses [88].

In contrast, the second group of chemokine-mediated genes showed significantly higher transcript abundance in ATT compared to ADJ (with GO annotation chemokine-mediated signaling pathway (GO:0070098), segment 2 (Figure 2); Supplementary File S9): CXCR1 coordinates neutrophil trafficking by liganding with CXCL6 and CXCL8 in inflamed tissues [89]. Several LECT2 homologs have been identified from various fish species and demonstrated to have conserved roles as a chemoattractant. LECT2 was identified as an APP during bacterial infection in zebrafish [90] and presented an mRNA upregulation in a lice density-dependent manner in both wild and farmed Atlantic salmon skin at 24–26 dpi [91]. The disagreement in *lect2-a* mRNA expression in our study and that of Gallardi et al. [91] might be influenced by differences in investigated tissues and sampling time points. Human CXCL2 (MIP2) is chemotactic for polymorphonuclear leukocytes, especially for neutrophils, and transcribed at the sites of wound repair [92]. We noted an interesting expression pattern for *cxcl11* (upregulated in segment 2) and its receptor *cxcr3* (downregulated in segment 1, Figure 2; Supplementary File S1). In zebrafish, the CXCR3-CXCL11 axis mediates macrophage recruitment against bacterial infection [93]. IL1β is a pivotal proinflammatory cytokine with diverse physiological roles and its major contribution in regulating the inflammatory process is conserved in fish [94]. Despite its transcriptional upregulation in ATT compared to ADJ, the decoy receptor of IL1β (i.e., IL-1RII) was downregulated in ADJ compared to PRE (Supplementary File S1). Mammalian IL11 signaling plays a crucial role in thrombopoiesis [95] and we found local upregulation of *il11* in ATT compared to ADJ. Taken together, our data suggest that transmigration and trafficking of different immune cells in salmon fin are dramatically altered through the transcriptional modulation of cytokines and receptors at the site of infection.

#### 3.4.3. Complement Pathway

We documented coordinated suppression of components of the complement cascade (*c4a*, *cfh,* and *cfd*), receptors (*cd93*, *c3ar1*, and *c5ar1*) and some regulators (*cd59*) of complement activation in this study. As an integral part of the immune system, the complement pathway detects pathogens, alarms the defense system, and eliminates pathogens. Moreover, it serves as a bridge between adaptive and innate immunity and orchestrates acquired immune responses [96]. Receptors of C3a and C5a (potent chemotactic and pro-inflammatory) peptides are expressed by macrophages and shown to participate in signal modulation of the complement system [97]. Mammalian CD93 is a C1q-receptor and implicated with intracellular adhesion, phagocytosis, and inter-cellular interactions [98]. Downregulation of complement-related genes in skin during the lice-infection has been previously reported [24]. Collectively, our findings indicate a potential disruption in the routine function of complement apparatus. Conversely, a member of the C1q family (*c1ql2*; [99]) was transcriptionally upregulated in infected animals compared to PRE (Figure 8D), but its association with lice infection is unclear.

#### 3.4.4. Antiviral Responses

In the present microarray study, several viral-induced transcripts (e.g., *ifi44*, *ifit5*, *trim*s, and *rsad2*) were downregulated in ATT and ADJ fin of lice-infected animals compared to PRE (Table 2). In the context of lice-infection, suppressed antiviral responsive pathways were evident in anterior kidney and skin of different salmonid species [8,26,39]. Barker et al. [13] recently demonstrated decreased transcript levels of antiviral-effector proteins (e.g., *mx*, *mhc-Ib*, *galectin 9*, *trim16*, and *trim25*) in anterior kidney of salmon by lice infection. In addition, these authors found increased infectious salmon anemia viral load and virus-mediated mortality in *L. salmonis*-infected salmon [13]. These findings imply that antiviral response was compromised during the lice-infection.

#### 3.4.5. Miscellaneous Elements in Innate Immunity

A panel of genes involved in innate immunity was downregulated in ATT and ADJ compared to PRE (segment 1; *hdd11*, *cd99*, *c1qtnf6*) or upregulated in ATT compared to PRE (segment 2; *fgl1*, *camp*, *arg2*, *nlrc3*) (Table 2). HDD11, a protein of unknown function that was bacteria-induced in silkworm [100], showed remarkable transcriptional suppression (~25-fold) in the present study. Downregulation of *cd99* indicates that the regulation of lymphocyte adhesion [101] might be affected in chalimus-attached fins. CTRP6, encoded by *c1qtnf6*, induces IL10 and is considered to be anti-inflammatory [102,103]. While these modulated transcriptional patterns indicated a favorable environment for parasitism, the expression of some other genes suggested that salmon mount certain defense measures. For instance, FGL1 is an immunosuppressive ligand of the LAG3 receptor that plays a T cell inhibitory role [104]. The mRNA level of *fgl1* was significantly suppressed in our study (Table 2). Two *camp* gene copies were found in Atlantic salmon and rainbow trout [105]. In the present study, these two paralogs demonstrated distinct expression profiles. Based on microarray, both *camp* paralogs were upregulated in ATT compared to ADJ, however this was only confirmed by QPCR for *camp-a* (Figure 6 and Figure 8A,E). Mitochondrial expression of ARG2 regulates nitric oxide synthesis, and its transcript upregulation, as reported earlier in salmonid skin [39], was suggestive of M1 (classically activated) macrophage infiltration into the cutaneous region at chalimus-attachment sites. NLRC3, that plays inhibitory roles during inflammation by impeding NF-κB activation [106], was significantly upregulated at the mRNA level in response to lice in ATT and ADJ compared to PRE (Table 2). These results indicate that chalimus-attachment modulated various innate immune elements in salmon fin.

#### 3.4.6. Adaptive Immunity

Our microarray data identified different dysregulated elements of adaptive immunity, as well (e.g., two MH class I transcripts (*hlab* and *hlah*), *b2m*; Table 2). Downregulation of MH class I and II antigen genes at the lice-attachment site in skin was previously evidenced in salmonids [24,38,39]. Infiltrated MH class II+ cells in dermis and epidermis of lice-infected coho salmon, which demonstrated marked upregulation of *mh class II* in the skin, provided strong evidence for the contribution of MH -associated T cell-mediated immunity to the lice-resistance [39]. Additionally, the gene encoding B2M, a structural component of MHC I complex, was also found to be less abundant in ATT and ADJ compared to PRE, as observed in an earlier study [23].

Similarly, our data indicated decreased transcript levels of some B cell markers (*iglc3*, *fcer1g*) in infected fish. Different host–parasite models have earlier suggested that the immunosuppression caused by parasites is at least in part due to the decreased adaptive immune responses. During amoebic gill disease (AGD), MH class I pathway-related genes were downregulated in *S. salar* [107]. Suppression of *mh class II* mRNA was also observed in carp infected with *Trypanoplasma borreli* [108]. During the bi-phasic defense response in Atlantic salmon against sea lice, elevated levels of *igm* and *igt* transcripts in skin occurred only at mid- (2 weeks post-infection) and later-stages, suggesting impaired B cell-mediated immunity during the early stage of infection [24].

### 3.5. Relationship Between Transcript Expression and Lice Load

A clear separation between control and lice-infected groups was observed in the PCoA and hierarchical clustering analyses performed using either microarray or QPCR data. In contrast, ADJ and ATT groups showed overlap in the PCoA and a mixed distribution in clustering analyses (Figure 1C,D and Figure 11). Our results suggest that contemporaneous regulation of elements responsible for opposing immune regulation was a part of the anti-lice response. For instance, several transcripts encoding positive regulators of immunity (e.g., cytokines: *ccl17*, *ifna-a*; APP: *crp*; redox markers: *txn-b*, *prxl2a*, *prdx1a*) demonstrated a negative correlation with lice burden (Figure 11B). In contrast, transcript abundance of *cd83*, a well-known marker for mature dendritic cells and an immune-suppressive molecule [109], showed a significant positive correlation with lice load. These observations confirmed that lice-induced immune suppression in fin is one of the strategies that could negatively impact the susceptible hosts.

### 3.6. Compromised Immune System by Lice

In resistant species (coho and pink salmon), skin responses against sea lice infection are characterized by filamentous cell proliferation, moderately increased intracellular space, abundant leukocyte infiltration, epidermal thickening, and hyperplasia [36,64]. The magnitude of both inflammatory and hyperplasic responses was postulated as the primary determinant of resistant versus susceptible traits against sea lice infection in salmonids [10]. In addition, it has been found that excretory and secretory products of sea lice that contains immunomodulatory substances (e.g., trypsins and prostaglandins) could also have regulatory potential on host immunity [110]. Data presented in our current study provide evidence for hypo-inflammatory responses, weak APR, and compromised immunity by transcriptomic suppression of several components of host defense, such as antiviral responses, wound repair mechanisms, immune signaling pathways, and redox homeostasis during lice infection.

In an experimental infection trial, all the parasites will likely be in similar/closer developmental stage(s) of their life cycle and potentially demonstrate a development stage-specific impact on their host. However, in the natural environment, the infection dynamics will be more complex, with multiple life-stages of sea lice potentially parasitizing the host. The host-response would also likely be more complicated and depend on multiple factors. With this in mind, chronic lesions and open wounds resulting from lice infection and feeding could act as ‘ports of entry’ to potential secondary pathogens. In addition, the potential of *L. salmonis* as mechanical vectors in transmitting bacterial and viral diseases has already been experimentally demonstrated [111,112]. This phenomenon adds an extra layer of complexity to the host–pathogen interactions and opens a new paradigm, namely co-infection, which has been insufficiently studied until recently [13]. A compromised host immune system resulting from sea lice infection could be the main cause of detrimental consequences of co-infection [13]. Hence, exploring potential control measures (e.g., modulating host immunity using novel feed formulations [14]) to tackle sea lice infections, and potential secondary infectious outbreaks, requires further research attention. Molecular biomarkers identified herein will be valuable tools in our future endeavors.

## 4. Materials and Methods

### 4.1. Ethics Statement

All procedures involving fish handling, treatment, euthanasia, and dissection were performed in accordance with the guidelines of the Canadian Council of Animal Care (approved Memorial University Institutional Animal Care Protocol 17-77-MR, May 2, 2017).

### 4.2. Experimental Animals

Salmon smolts were purchased from a regional farm (Stephenville, NL, Canada) and transported to the Dr. Joe Brown Aquatic Research Building (JBARB, Ocean Sciences Centre (OSC), Memorial University of Newfoundland (MUN), Canada), where they were transferred into 3800 L tanks. After arrival, salmon were intraperitoneally PIT (passive integrated transponder)-tagged. Eighty post-smolts (307.3 ± 27.8 g mean initial weight ± SE) were transferred to the bio-containment zone in the Cold-Ocean Deep-Sea Research Facility (CDRF, OSC, MUN) and randomly distributed to and maintained under a 24 h light photoperiod in two 620 L tanks (40 fish/tank and 27 ± 1.5 kg/m^3^) with a flow-through seawater supply. Fish were fed nightly with a commercial diet (Dynamic S, EWOS, Cargill) to satiation using automatic feeders. Fish were acclimated to laboratory conditions for four weeks before sea lice infection. Water quality parameters, such as temperature and oxygen saturation, were stably maintained throughout the experimental period (at 10 ± 1 °C and >90% O_2_ saturation; Supplementary Tables S1 and S2). Fish were starved for 24 h before any handling or sampling.

### 4.3. Sea Lice Infection and Sampling

Adult female *L. salmonis* sea lice were collected from marine aquaculture sites in the Bay of Fundy (St. Andrews, NB, Canada) and transported to the Huntsman Marine Science Centre (St. Andrews, NB, Canada). Egg-strings were removed from adult females and maintained in hatching chambers at 10–12 °C for 9–10 days until they developed to infective free-living copepodid stage lice, which were then transported to CDRF.

Just before the infection, the water supply for tanks was turned off, the water level was reduced by 50%, and water was oxygenated with air diffusers to maintain ~100% O_2_ saturation at 10 ± 1 °C. Atlantic salmon were then bath-infected with *L. salmonis* by adding ~50 copepodids per fish to each tank. Water quality parameters (dissolved O_2_ and temperature) were continuously monitored throughout the infection period at intervals of 10 min. Water supply was reinstated after 2 h of copepodid exposure. At least five individuals from each tank at 0 days post-infection (dpi; prior to infection, PRE), 8 dpi, and 30 dpi (when most lice were at their chalimus and pre-adult stages, respectively) were euthanized with a lethal dose of MS-222 (400 mg/L; Syndel Laboratories, BC, Canada) and dissected for sample collection (Figure 12A). Parasites on each salmon were counted. Samples from pelvic fin tips at an area of the fin with lice ‘attached’ (ATT) and ‘adjacent’ to chalimus attachment sites with no signs of previous lice attachment (ADJ) were excised as shown in Figure 12B, chalimi were removed from ATT sites using sterile forceps, and fin samples were flash-frozen in liquid nitrogen and stored at −80 °C until RNA extraction was performed.

### 4.4. RNA Extraction, DNA Digestion, and Column Purification

Fin tips were homogenized in TRIzol^®^ Reagent (Invitrogen/Life Technologies, Burlington, Canada) using a TissueLyser II (QIAGEN, Restch, GmbH, Germany), and total RNA was extracted from the homogenate as per the manufacturer’s instructions. To eliminate any residual genomic DNA contamination, 40 µg of crude RNA from each sample was treated with DNase I (6.8 Kunitz units; RNase-Free DNase Set, QIAGEN) and then column-purified by using the RNeasy Mini Kit (QIAGEN) following the manufacturer’s protocol. The RNA concentration and purity were assessed by ND-1000 UV spectrophotometry (NanoDrop, Wilmington, DE, USA), and the RNA integrity was examined by 1% agarose gel electrophoresis. The samples with tight 18S and 28S ribosomal RNA bands and high A260/280 and A260/230 ratios (>1.8) were used in transcriptional analyses.

### 4.5. Microarray Experiment: Design, Hybridization, and Data Acquisition

The microarray experiment was designed and conducted in compliance with the MIAME (Minimum Information About a Microarray Experiment) guidelines [113]. Fin RNA samples of six fish at 8 dpi (three from each duplicate tank) each from control (PRE) and sea lice-infected salmon (both ATT and ADJ samples collected from the same fish), selected based on RNA quality, were used in the microarray experiment (i.e., 12 animals and 18 samples in total). The microarray study was based on a common reference design (Figure 12C), in which the differences among sample groups in fin transcriptome were investigated by contrasting individual samples against a common reference pool prepared from the equal contribution of all 18 samples. A consortium for Genomic Research on All Salmonids Project (cGRASP)-designed 44K Atlantic salmon oligonucleotide array (GEO accession: GPL11299; Agilent Technologies, Mississauga, Canada) that comprises 60mer probes was used as the microarray platform [27].

The antisense amplified RNA (aRNA) preparation and labeling were conducted using the Amino Allyl MessageAmp II aRNA amplification kit (Invitrogen). One microgram of DNase I-treated, column-purified total RNA was reverse-transcribed to the first-strand cDNA and used in second-strand cDNA synthesis. The double-stranded cDNA was then used to synthesize modified aRNA using the manufacturer’s instructions. The aRNA samples were column-purified and subjected to the quantity and quality assessment by NanoDrop spectrophotometry and agarose gel electrophoresis, respectively. A common reference was prepared by pooling 10 µg of each of the 18 experimental samples. Twenty micrograms of each experimental and common reference aRNA sample were precipitated overnight through standard molecular biology methods and then resuspended in coupling buffer. While 18 individual experimental aRNA samples were labeled with Cy5, the common reference was labeled with Cy3 (GE Healthcare, Mississauga, ON, Canada) through a dye-coupling reaction, as per the manufacturer’s instructions. The labeling efficiency was determined using the ‘microarray’ mode in the ND-1000 NanoDrop spectrophotometry. For each array, equal amounts (825 ng) of each Cy5-labelled experimental aRNA and Cy3-labelled reference aRNA were pooled, fragmented following the manufacturer’s protocol (Gene Expression Hybridization Kit; Agilent, Santa Clara, CA, USA), and co-hybridized to a 44K salmonid oligonucleotide microarray. Each array was hybridized at 65 °C for ~17 h at a 10 rpm rotation in an Agilent hybridization oven. Array slides were then washed with Gene Expression Wash Buffer 1 and 2 (Agilent) according to the manufacturer’s guidelines, and residual wash buffer was removed by centrifuging at 200× *g* for 5 min at room temperature.

Each microarray was scanned at 5 µm resolution using a SureScan D Microarray Scanner (G2600D, Agilent Technologies) using Agilent Scan Control Software (v9.1.11.7, Agilent Technologies) by applying a built-in protocol (Agilent_HD_GX_2color). Photomultiplier tube (PMT) sensitivity for Cy3 and Cy5 dye channels was set to 100%. The resulting TIFF images (20 bit) containing raw array data were then subjected to Agilent Feature Extraction (FE, v12.0.3.1) software to retrieve the signal intensity data of each microarray probe using a protocol provided by the developer (GE2_1200_June14). Probe signal, background signal subtraction, LOWESS normalization, outlier flagging, and log_2_-ratio calculations were performed by FE. Quality control (QC) parameters from QC reports of each array generated by FE were also examined. Resulting text files containing raw microarray data were then subjected to GeneSpring GX, a multi-omic analysis platform (v14.9.1, Agilent Technologies), to visualize and further process the data. The missing values were imputed using a method embedded within GeneSpring GX. Based on QC assessment and downstream analyses (such as heatmaps and principal component analyses (PCA)), samples considered as potential outliers were identified (1 fish/group) and excluded from the downstream analyses. Features absent in more than 25% of the arrays in all three groups (PRE, ADJ, and ATT) were discarded, resulting in a final list that consisted of 28,065 probes for statistical analyses. Microarray data from this study have been submitted to Gene Expression Omnibus (GEO) under the accession GSE140756.

### 4.6. Microarray Data Analysis

The DEPs between sample groups (PRE, ADJ, and ATT) were determined by Significance Analysis of Microarrays (SAM) [31] using the Bioconductor package siggenes in the R environment (v1.1.463). Two-class comparison with a false discovery rate (FDR) cutoff of 0.05 or 0.01 was performed to identify the chalimus-responsive probes between groups (i.e., ATT versus PRE, ADJ versus PRE, and ATT versus ADJ). The resulting transcript list identifications were updated using the contiguous sequences (contigs) or expressed sequence tags (ESTs) that were used to design the 60mer probes of the array [27]. BLASTx searches of these transcript sequences against the NCBI non-redundant amino acid (nr) and Swiss-Prot databases were performed with default settings (E-value threshold < 1 × 10^−5^). The resulting BLASTx hits were mapped to gene ontology (GO) terms of chalimus-responsive transcripts in each sample group (biological process (BP), molecular function (MF), cellular component (CC)) using the Blast2GO program (BioBam Bioinformatics, Valencia, Spain) [114].

The annotation assessment of this salmonid 44K 60mer oligo microarray resulted in >10K probes with “unknown” annotations. In order to update the annotation, 60mer probes and their representative contig sequences were searched against the Swiss-Prot database (April 2019 version) and the NCBI nr/nt database. The resulting hits were filtered with E-value < 1 × 10^−5^, identity percentage (>75%) and query coverage percentage (>50%). The filtering criteria for 60mer probe BLASTn hits were stringent (only 2 allowed mismatches with un-gapped alignment option). The probe annotations were also revised by homology sequence searches in updated genome annotation databases for *S. salar*, *O. mykiss*, *Danio rerio*, and *Homo sapiens*. The gene symbols for probes were assigned from HUGO Gene Nomenclature Committee (HGNC; https://www.genenames.org/) and/or GeneCards (https://www.genecards.org/) databases.

### 4.7. GO Term Enrichment Test, Analyses and Visualization of GO/Pathway Term Networks and Expression Profiles

Initially, a GO term enrichment test was performed on DEP lists, which were subjected to a fold-change (FC) cutoff of |2|, by using the Blast2GO (Fisher’s Exact Test, FDR cutoff of 0.01) with the 44K salmon array as the reference set. Secondly, the functional implications of the lists of differentially expressed genes (DEGs) were examined using ClueGO [32] plugin in Cytoscape (v3.5.1) [33]. The GO databases (23.05.2019) for BP, MF, and CC, and the Reactome pathways database (23.05.2019) were used. The enrichment/depletion analysis was performed using a two-sided hypergeometric test after its adjustment by the Bonferroni step-down procedure. The GO term fusion strategy was employed to integrate GO categories, minimize the complexity, and create a functionally organized GO cluster network. The leading terms were ranked based on their significance with different *p*-values for different gene sets with Kappa-statistics score threshold set to 0.4.

We performed a Pathifier pathway analysis that incorporated transcriptional expression data to infer dysregulated pathways. All the significant GO/pathway terms from the above section were pooled and their Pathway Deregulation Score (PDS) based on expression was computed by using Pathifier Bioconductor package 1.16.0 in the R environment. This method by Drier et al. [34] calculates the PDS for each pathway per sample by using the annotation resources such as the MSigDB (http://software.broadinstitute.org/gsea/msigdb. Using the C5 collection of MSigDB, 134 GO/pathway terms were annotated and the PDS was estimated for infected salmon (ADJ and ATT) with respect to the non-infected control (PRE).

Pearson correlation and complete linkage clustering functions in the Genesis program were used in generating the hierarchical clustering and heatmaps of median-centered data of DEPs [115]. Alternatively, unsupervised clustering on Euclidean distance of PDS values was constructed using Ward’s agglomerative linkage method (ward.D2) and visualized as a heatmap by hierarchical clustering using the heatmap3 R package [116].

### 4.8. Real-Time Quantitative Polymerase Chain Reaction (QPCR) Confirmation

Thirty-one microarray-identified GOIs, including some paralogs for 5 GOIs, were selected from the gene lists for transcriptional profiling by QPCR. GOIs were selected based on the functional themes they represented (i.e., ‘ECM catabolism, tissue remodeling, and wound healing’, ‘immunity and defense’, ‘chemotaxis and signaling’, ‘antiviral response’, and ‘redox homeostasis’; Supplementary Table S3). Another member of the MMP family (*mmp20*), which was not identified by the microarray analyses, was also added to the QPCR analysis.

In addition to the samples used in the microarray experiment, we included 4 more samples from each group (PRE (10 fish), ADJ and ATT (10 fish)) in the QPCR-confirmation experiment (*n* = 10/group; 30 samples in total). First-strand cDNA templates for QPCR were synthesized in 20 μL reactions from 1 μg of DNaseI-treated, column-purified total RNA using random primers (250 ng) and M-MLV reverse transcriptase (200 U; Invitrogen/Life Technologies) with the manufacturer’s first strand buffer (1× final concentration), dNTPs (0.5 mM final concentration), and DTT (10 mM final concentration) at 37 °C for 50 min. The resulting cDNA was diluted either 20 times (for QC; see below) or 40 times (for normalizer testing and expression study) with nuclease-free water (Invitrogen). QPCR assays were performed in technical duplicates (for QC) or triplicates (for normalizer testing and expression study) using Power SYBR Green I dye chemistry in 384-well format on a ViiA 7 Real-Time PCR system (Applied Biosystems, Thermo Fisher Scientific). Each QPCR plate included a no-template control (NTC). Each assay was performed in 13 µL reaction containing 1× Power SYBR Green PCR Master Mix (Applied Biosystems/Life Technologies), 50 nM of both the forward and reverse primers, and 4 μL of diluted cDNA (5 ng input total RNA for expression study and normalizer testing, and a serially-diluted RNA for amplification efficiency determination; see below). The QPCR profile consisted of 1 cycle of 50 °C for 2 min, 1 cycle of 95 °C for 10 min, and 40 cycles of 95 °C for 15 s and 60 °C for 1 min, with fluorescence detection at the end of each 60 °C step.

Details of each QPCR assay, such as primer sequences, amplification efficiency, and amplicon size, are presented in Supplementary Table S3. BLASTn searches against each GOI were performed using nr/nt and EST databases of NCBI in order to identify any existing paralogs. By aligning the sequences of identified putative paralogs in Vector NTI (Vector NTI Advance 11, Life Technologies), potential regions (i.e., with at least 2 bp difference) for designing paralog-specific primers were identified. Primers were designed using either Primer 3 (v4.1.0; http://bioinfo.ut.ee/primer3/) or PrimerQuest (Integrated DNA Technologies, Inc.; https://www.idtdna.com/Primerquest/Home/Index). Prior to performing QPCR assays, each primer pair underwent a series of QC procedures. In brief, a reference RNA pool was prepared using four randomly selected RNA samples from each sample group (12 in total). Amplification efficiencies [117] were determined from the standard curves generated using a 5-point 1:3 dilution series starting with cDNA corresponding to 10 ng of input total RNA. It was ensured that each primer pair demonstrated a single peak in the dissociation curve analysis and had an amplification efficiency > 90% with no amplification in NTCs.

Eight candidate normalizer transcripts were evaluated: *actb* (*β-actin*), *rpl32* (*60S ribosomal protein 32*), two paralogs of *ef1a* (*elongation factor 1α*), *abcf2* (*ATP binding cassette sub-family F member 2*), *pabpc1* (*polyadenylate-binding protein 1*), *eif3d* (*eukaryotic translation initiation factor 3 subunit D*), and *ndufs7* (*NADH dehydrogenase (ubiquinone) iron-sulfur protein 7*). Primer pairs for these candidate normalizers were designed and quality tested as described above. Template cDNA of each sample (corresponding to 5 ng of total input RNA) was used to measure the fluorescence threshold cycle (C_T_) against each candidate normalizer and then subjected to geNorm analyses (qBASE plus, Biogazelle NV, Belgium) [118]. Among the normalizers, based on geNorm M-value, ef1a1 (M = 0.165) and eif3d (M = 0.173) were chosen as reference genes for the QPCR analyses.

After completing the primer QC and normalizer test, QPCR analyses were performed in compliance with MIQE (Minimum Information for Publication of Quantitative Real-Time PCR Experiments) guidelines [119] to determine the transcript expression of GOIs. The relative quantity (RQ) of each transcript was determined by a qBase relative quantification framework [120,121] by using the C_T_ values measured for GOIs and reference genes by the ViiA 7 Software (v1.2.3; Applied Biosystems, Thermo Fisher Scientific). RQ of each GOI was calculated by normalizing against transcript levels of both *ef1a1* and *eif3d*, and by incorporating the amplification efficiencies. The individual with the lowest normalized expression level was considered as the calibrator (i.e., RQ = 1) for each GOI.

### 4.9. Statistical Analyses

RQs of GOIs were checked for outliers using Grubb’s test, and for homoscedasticity using Levene’s test. In total, 21 RQ values were identified as statistical outliers in the entire dataset (i.e., out of 1110 RQ values), and were excluded from the study. The differences among treatments were then compared using one-way ANOVA followed by Tukey’s post hoc test. The accepted level of significance was *p* < 0.05. All the statistical analyses above were conducted using IBM SPSS Statistics (v25.0.0; IBM Corp, Armonk, NY).

FC values derived from microarray and QPCR were log_2_-transformed and analyzed for correlation via linear regression. A significant correlation between both datasets was considered as evidence for the validity of the microarray results.

Normalized log_2_ ratios from microarray data were transformed (by adding a constant so that values were positive) and analyzed using Principal Coordinates Analysis (PCoA) for similarities among the expression patterns of DEPs observed among the salmon. Similarly, standardized RQ values of GOIs from QPCR were used along with phenotypic parameters (i.e., chalimus count, weight, length, hepatosomatic index (HSI), spleen-somatic index (SSI), viscerosomatic index (VSI), and condition factor (K)) in PCoA. We also tested for differences among gene expression patterns via permutational multivariate analysis of variance (PERMANOVA) with 9999 random permutations. The accepted level of significance was *p* < 0.05. Both PERMANOVA and PCoA were based on Bray–Curtis similarities of all pairwise comparisons among individuals. These analyses were performed using PRIMER 6.1.15 (Ivybridge, UK). The complete linkage hierarchical clustering was conducted with PRIMER using Pearson correlation resemblance matrices. Log_2_-transformed phenotypic and QPCR-gene expression data were subjected to Pearson correlation analyses using IBM SPSS to find any relationship between them, and the resulting Pearson correlation coefficients (r) were tabulated as a correlation matrix.

## 5. Conclusions

Using a laboratory infection model and molecular techniques (i.e., microarray and QPCR), we investigated and profiled the transcriptome response in fin tissue at the early (chalimus) stage of sea lice infection in Atlantic salmon. Findings from the current study significantly contribute to the understanding of the physiological basis for Atlantic salmon’s high vulnerability to sea lice infection, particularly at the chalimus stage. Immunosuppression at transcriptional levels of several GOIs in ATT and ADJ sites compared to PRE suggested lice-associated immunomodulation, by which chalimus disrupts or evades the defense strategies of the host. A comparison between ATT and ADJ sites, however, indicated that Atlantic salmon attempt to mount a local anti-parasite response at the site of chalimus-attachment. Transcriptional changes that were documented in this study in response to chalimus infection on fin tissue may help to explain the Atlantic salmon’s inability to mount a tissue response against sea lice that is sufficiently robust to expel the parasite.

## Figures and Tables

**Table 1:** Selected transcripts playing roles in different physiological processes (except those functioning primarily in immunity) and their fold-change values from SAM.

Category	Probe ID (44K) ^1^	Gene Symbol_v1 ^2^	Gene Description ^3^	Fold-Changes ^4^			Segment ^5^
				ADJ versus PRE	ATT versus PRE	ATT versus ADJ	(Figure 2)
**Transcription factors**	C090R002	*hoxd12*	Homeobox protein Hox-D12a-like	−13.7	−14.3	NS	1
	C180R160	*hoxd10*	Homeobox protein Hox-D10	−3.1	−2.8	NS	1
	C072R152	*hoxa9*	Homeobox protein Hox-A9b-like	−2.6	−2.0	NS	1
	C191R067	*hoxc11*	Homeobox protein Hox-A11a-like	−2.2	−2.2	NS	1
	C072R116_4_	*hoxc9*	Homeobox protein Hox-C9	2.9	2.6	NS	1
	C032R060	*hoxa5*	Homeobox protein Hox-A5	3.1	2.7	NS	1
	C033R115	*hoxc6*	Homeobox protein Hox-C6-like	3.1	3.0	NS	1
	C190R162_2_	cebpb*	CCAAT/enhancer-binding protein beta (C/EBP beta)	NS	NS	2.0	2
	C262R105	*gfi1b*	Zinc finger protein Gfi−1b	7.0	22.4	NS	1
	C088R057	*ppard*	Peroxisome proliferator activated receptor delta	NS	29.9	8.7	2
**Sugar and lipid metabolism**	C149R170_4_	*cox6b1*	Cytochrome c oxidase subunit 6B1	−3.8	−3.0	NS	1
	C201R100_2_	*tkt*	Transketolase	2.3	1.9	NS	1
	C255R056	*g6pc*	Glucose-6-phosphatase	1.3	2.2	1.6	1
	C228R118	*aldoc*	Fructose-bisphosphate aldolase C	4.3	8.2	NS	1
	C120R022	*nd1*	NADH dehydrogenase subunit 1	2.9	3.1	NS	1
	C150R058	*mettl17*	Methyltransferase-like protein 17	3.9	9.4	NS	1
	C138R121_3_	*gapdh*	Glyceraldehyde-3-phosphate dehydrogenase	2.5	2.9	NS	1
	C217R012	*aloxe3*	Arachidonate Lipoxygenase 3 (Hydroperoxide isomerase)	5.9	16.7	NS	1
**Melanin biosynthesis**	C178R076	*dct*	L-dopachrome tautomerase-like	−20.9	−18.2	NS	1
	C218R159	*tyrp1*	5,6-dihydroxyindole-2-carboxylic acid oxidase	−11.8	−13.1	NS	1
	C217R115	*tyr*	Tyrosinase	−9.9	−11.2	NS	1
**ECM degradation, tissue**	C184R018_7_	*col10a1*	Collagen alpha-1(X) chain	−4.2	−4.1	NS	1
	C199R006	*col6a1*	Collagen alpha-1(VI) chain	−2.8	−2.6	NS	1
**repair/remodeling, and wound**	C114R049_2_	*col15a1*	Collagen alpha-1(XV) chain	−2.2	−2.2	NS	1
**healing**	C095R156	*lamc1*	Laminin subunit gamma-1	−3.9	NS	NS	3
	C184R030_2_	fn1*	Fibronectin	−3.7	−1.8	2.0	1,2
	C170R165	bgn-a*	Biglycan paralog a	−2.5	−2.6	NS	1
	C237R076	bgn-b*	Biglycan paralog b	−4.6	−3.6	NS	1
	C222R057	*dcn*	Decorin	−1.8	−2.4	NS	1
	C265R134_2_	*htra1*	Serine protease HTRA1	−2.2	−2.1	NS	1
	C240R103	*htra3*	Serine protease HTRA3	−2.1	−1.8	NS	1
	C176R151_4_	*ctsb*	Cathepsin B	−2.0	NS	NS	3
	C200R137_8_	*anxa13*	Annexin A13	−2.4	−2.4	NS	1
	C143R165_5_	*anxa2*	Annexin A2	−2.4	NS	NS	3
	C185R098_3_	*serpinh1*	47 kDa HSP/collagen-binding protein	−2.3	−2.2	NS	1
	C015R046	*itga10*	Integrin alpha-10	−2.6	NS	NS	3
	C080R081_2_	*tgfbi*	Transforming growth factor-beta-induced protein	−2.0	−1.9	NS	1
	C205R135_2_	*ctgf*	Connective tissue growth factor (CCN family member 2)	−2.1	NS	NS	3
	C098R103	*fgf1*	Fibroblast growth factor 1	−11.8	NS	5.0	2
	C081R049	mmp9-a*	Matrix metalloproteinase-9 paralog a	−2.5	NS	2.2	2
	C047R004	mmp9-b*	Matrix metalloproteinase-9 paralog b	−2.2	NS	NS	3
	C137R132	mmp13-d*	Matrix metalloproteinase-13 (Collagenase 3) paralog d	−3.0	−1.9	NS	1
	C245R152_3_	mmp13-b*	Matrix metalloproteinase-13 (Collagenase 3) paralog b	−5.2	NS	3.3	2
	C133R147	mmp13-c*	Matrix metalloproteinase-13 (Collagenase 3) paralog c	NS	NS	2.9	2
	C048R162_8_	mmp2*	Matrix metalloproteinase-2	−2.3	−2.3	NS	1
	C062R075	mmp19*	Matrix metalloproteinase-19	−3.2	−2.1	NS	1
	C099R050	mmp23b*	Matrix metalloproteinase-23b	−2.1	−1.6	NS	1
	C246R156_3_	timp2-b*	Metalloproteinase inhibitor 2 paralog b	−2.7	NS	1.9	2
	C242R139	timp2-c*	Metalloproteinase inhibitor 2 paralog c	−2.4	NS	NS	3
	C135R154	*timp3*	Metalloproteinase inhibitor 3	−3.9	−2.5	NS	1, 3
**Oxygen transport**	C220R134_4_	*hba/hba4*	Hemoglobin subunit alpha (and -4)	−2.9	−2.6	NS	1
	C171R164_21_	*hbb*	Hemoglobin subunit beta	−3.2	−3.0	NS	1
	C088R086_5_	*alas2*	5-aminolevulinate synthase	−2.5	−2.3	NS	1
	C255R103_2_	*cygb*	Cytoglobin-2	2.0	NS	NS	3
**Redox homeostasis**	C104R146	*txndc5*	Thioredoxin domain-containing protein 5	−2.4	−1.8	NS	1
	C264R109_2_	prxl2a*	Redox-regulatory protein fam213a (peroxiredoxin-like 2A)	−6.4	−6.7	NS	1
	C174R143_2_	gpx7*	Glutathione peroxidase 7	−2.9	−3.1	NS	1
	C210R126_4_	gsta*	Glutathione S-transferase A-like	−2.6	−2.1	NS	1
	C115R114_6_	ncf2*	Neutrophil cytosol factor 2-like isoform X2	−1.9	−1.6	NS	1
	C211R125_3_	txn-b*	Thioredoxin paralog b	−2.7	NS	2.2	2, 3
	C115R114_5_	prdx1-a*	Peroxiredoxin 1 paralog a	−1.7	NS	NS	3
	C198R137	*fth1*	Ferritin heavy chain	−1.6	NS	NS	3
	C123R075_2_	*frim* ^†^	Ferritin, middle subunit	−2.7	NS	NS	3

^1^ Identifier of the probe on the 44K array; if a transcript is represented by multiple probes, fold-change (FC) values are presented as mean FC values of all probes and the number of probes contributing to the mean FC is indicated by a subscript number next to the ID. ^2^ Official gene symbols (version 1) are based on multiple annotations and majority of them are represented in HGNC (https://www.genenames.org/) and/or GeneCard (https://www.genecards.org/) databases. *, transcripts that were QPCR-assayed. †, middle subunit of ferritin is not found in mammals and a putative symbol is used (alias *ftm*). ^3^ Name(s) or alias(es) obtained from annotation. Refer the Supplementary File S1 for additional details. ^4^ FC in three different comparisons for differentially expressed probes (DEPs; false discovery rate < 0.05) obtained from Significant Analysis of Microarray (SAM). Fold downregulation was calculated as the inverse of FC (i.e., -1/FC) for the original values that were less than one in the SAM output. If a probe is absent in a particular comparison, the corresponding FC is indicated by NS (not significant). ADJ versus PRE, ATT versus PRE, and ATT versus ADJ indicate the FC of first treatment group with respect to the second. Most of the DEGs presented here possess FC ≥ 2 and are biologically relevant to sea lice infection. ^5^ In which segment (refer to Figure 2) of the microarray DEP list a particular transcript is present.

**Table 2:** Selected transcripts associated primarily with different components of the immune system and their fold-change values from SAM.

Category	Probe ID (44K) ^1^	Gene Symbol_v1 ^2^	Gene Description ^3^	Fold-Changes ^4^			Segment ^5^
				ADJ versus PRE	ATT versus PRE	ATT versus ADJ	(Figure 2)
**Pattern recognition**	C188R074_5_	*cd93*	Complement component C1q receptor	−2.1	−1.7	NS	1
**receptors (PRRs)**	C084R109_2_	*cd209*	CD209 antigen	−1.9	−1.8	NS	1
	C126R092	*cd302*	CD302 molecule	−2.1	−2.0	NS	1, 4
	C220R158_2_	*mbl2*	Mannose-binding protein C	−2.4	−2.4	NS	1
	C001R135_3_	*mrc1*	Macrophage mannose receptor 1	−1.8	NS	NS	3
	C117R009	*clec4m*	C-type lectin domain family 4 member M (CD209-like)	−2.0	NS	NS	3
	C103R112	*clec4e*	C-type lectin domain family 4 member E	−2.1	NS	2.6	2, 1
	C089R099_10_	lgals1*	Galectin-1	−2.9	−3.2	NS	1
**Cytokine/chemokine**	C180R124	*ccl4*	C-C motif chemokine 4	−6.5	−4.3	NS	1
**signaling**	C203R129	*tgfb3*	Transforming growth factor beta-3	NS	−1.6	NS	4
	C185R165_4_	*cmklr1*	Chemokine-like receptor 1	−3.4	−2.8	NS	1
	C095R021	ccl17*	C-C motif chemokine 17-like	−1.6	NS	NS	3
	C111R068	*il1r2*	Interleukin-1 receptor type 2	−2.4	NS	NS	3
	C252R096_2_	*ccr4*	C-C chemokine receptor type 4	−1.8	NS	NS	3
	C159R150_2_	*il13ra2*	Interleukin-13 receptor subunit alpha-2	3.1	3.0	NS	1
	C202R162	ccrl1*	Chemokine receptor 4-like	2.3	1.9	NS	1
	C162R124	*cxcr1*	C-X-C chemokine receptor type 1	−5.3	NS	2.5	2
	C159R112_2_	lect2-a*	Leukocyte cell-derived chemotaxin-2 paralog a	−8.2	NS	2.4	2
	C133R130	cxcl11*	C-X-C motif chemokine 11-like	−4.4	NS	2.0	2
	C183R028_2_	cxcl2-b*	C-X-C motif chemokine 2 (MIP2) paralog b	NS	NS	3.1	2
	C230R100	*il1b*	Interleukin-1 beta	NS	NS	2.2	2
	C197R010	*il11*	Interleukin 11	NS	NS	3.7	2
**Complement system**	C070R162_5_	*cfh*	Complement factor H	−2.9	−2.8	NS	3, 1
	C195R122_3_	cd59*	CD59 glycoprotein	−2.9	−2.4	NS	1
	C232R083_2_	*c4a*	Complement C4	−3.0	−2.2	NS	1
	C184R062	*cfd*	Complement factor D	NS	−2.2	NS	4
	C153R129	*c3ar1*	C3a anaphylatoxin chemotactic receptor	−2.6	−2.2	NS	1
	C073R014_2_	*c5ar1*	C5a anaphylatoxin chemotactic receptor 1	−1.7	NS	NS	3
	C191R156	*cfb*	Complement factor B	2.2	2.1	NS	1
**Coagulation cascade**	C089R005	*f3*	Coagulation factor III (tissue factor)	−3.9	−2.3	NS	1
*(Some of these also take part in acute phase response (APR))*	C009R078_3_	*tfpi2*	Tissue factor pathway inhibitor 2 precursor	−3.3	−2.8	NS	1
	C013R163	*f10*	Coagulation factor X	2.1	1.7	NS	1
	C137R137_3_	f5*	Coagulation factor V	NS	NS	2.5	2
	C127R168	*serpine1*	Plasminogen activator inhibitor 1	−5.5	NS	2.3	2
**Inflammation and**	C145R001	crp*	C-reactive protein	−2.2	−2.6	NS	1
**acute phase**	C068R154_2_	*mdk*	Midkine	−2.3	−1.8	NS	1
**response (APR)**	C254R103	*ptges*	Prostaglandin E synthase	−2.0	−1.7	NS	1
	C042R087_3_	*vcam1*	Vascular cell adhesion protein 1	−2.4	−1.6	NS	1
	C003R128	*gpr44*	Prostaglandin D2 receptor 2	2.3	NS	NS	3
**Antiviral response**	C139R032	rsad2-a*	Radical S-adenosyl methionine domain-containing protein 2 (Viperin) paralog a	−2.3	−2.7	NS	1
	C080R031_3_	*ifi44*	Interferon-induced protein 44 (p44) (Microtubule-associated protein 44)	−1.8	−2.3	NS	1,4
	C055R128	*ifit5*	Interferon-induced protein with tetratricopeptide repeats 5	−2.3	−2.3	NS	1
	C218R011	ifna-a*	Interferon a3 paralog a	2.2	2.3	NS	1
	C168R125	*trim8*	E3 ubiquitin-protein ligase TRIM8	−1.8	−1.8	NS	1
	C184R150	*trim58*	Zinc-binding protein A33	−2.4	−2.1	NS	1
**Innate immunity**	C024R014	hdd11*	Putative defense protein Hdd11	−24.9	−24.6	NS	1
**(general)**	C164R125_3_	*cd99*	CD99 antigen-like protein 2	−2.4	−2.7	NS	1
	C029R116_2_	cd83-a*	CD83 antigen paralog a	−1.7	−1.7	NS	1
	C200R158	*c1qtnf6*	Complement C1q tumor necrosis factor-related protein 6	−2.3	−2.5	NS	1
	C230R128	*lbp*	Lipopolysaccharide-binding protein (LBP)	−1.9	−1.9	NS	1
	C092R099_7_	*litaf*	Lipopolysaccharide-induced tumor necrosis factor-alpha	−1.7	−1.8	NS	1
	C107R123_3_	*c1qbp*	Complement component 1 Q subcomponent-binding protein, mitochondrial	−1.9	NS	NS	3
	C121R047	*tnfrsf11b*	Tumor necrosis factor receptor superfamily member 11B	NS	−2.1	NS	4
	C076R108	*fgl1*	Fibrinogen-like protein 1	−3.9	−2.4	NS	1
	C234R046	camp-a*	Cathelicidin antimicrobial peptide paralog a	NS	2.9	4.0	2
	C222R090	camp-b*	Cathelicidin antimicrobial peptide paralog b	−2.7	NS	2.9	2
	C098R103	*fgf1*	Fibroblast growth factor 1	−11.8	NS	5.0	2
	C138R013	*arg2*	Arginase-2	NS	NS	1.9	2
	C249R133_2_	c1ql2*	Complement C1q-like protein 2	NS	10.3	NS	4
	C234R042_5_	*nlrc3*	NLR family CARD domain-containing protein 3	1.7	2.2	NS	3, 1
	C095R050	c1qtnf3*	Complement C1q tumor necrosis factor-related protein 3	4.1	12.0	NS	1, 4
**Adaptive immunity**	C243R111	*hlab*	HLA (MHC) class I histocompatibility antigen, B-51 alpha chain	−2.3	−2.6	NS	1
	C211R164	*hlah*	HLA (MHC) class I histocompatibility antigen, alpha chain H	−3.7	−3.8	NS	1
	C141R114_18_	*b2m*	Beta-2-microglobulin	−1.6	−1.7	NS	1
	C263R031	*iglc3*	Immunoglobulin lambda constant 3	−3.2	−3.8	NS	1
	C250R1447	*fcer1g*	High affinity immunoglobulin epsilon receptor subunit gamma	−1.7	−1.7	NS	1
	C229R169_6_	*loc106606767*	Immunoglobulin mu chain C region	−5.0	NS	NS	3

^1−5^ Refer to Table 1 for details on all column constructions.

**Table 3:** Summary of details about the real-time quantitative polymerase chain reaction (QPCR)-analyzed genes of interest (GOIs) from different functional themes.

Functional Theme ^1^	Gene Description ^2^	Gene Symbol_v1 ^3^	General Function ^4^	Enriched GO Terms Associated ^5^	Segment in MA ^6^	Pathway Figure(s)
1. ECM degradation, tissue repair/remodeling and wound healing	TIMP metalloproteinase inhibitor 2	*timp2*	Modulating the activity of several MMPs	Activation of MMPs, Degradation of the ECM	2	Figure 5
	collagenase 3-like/matrix metalloproteinase-13	*mmp13*	ECM catabolism, cartilage degradation, tissue remodeling, wound healing	Activation of MMPs, Degradation of the ECM, Collagen degradation/catabolic process	2	Figure 4B, Figure 5
	matrix metalloproteinase 2 (72 kDa type IV collagenase)	*mmp2*	ECM catabolism, tissue repair/remodeling, inflammation	Extracellular space, ECM organization, Degradation of the ECM	1	Figure 4B
	matrix metalloproteinase-23	*mmp23b*	ECM catabolism	Extracellular space, ECM organization, Collagen-containing ECM	1	Figure 4B, Supp. Figure S2B
	matrix metalloproteinase-20 ^#^	*mmp20*	ECM catabolism	Not identified by SAM	-	-
	matrix metalloproteinase-19	*mmp19*	ECM catabolism	Extracellular space, ECM organization, Degradation of the ECM	1	Figure 4B
	matrix metalloproteinase-9	*mmp9*	ECM catabolism, leukocyte migration	Activation of MMPs, Degradation of the ECM, Collagen degradation/catabolic process, Regulation of inflammatory response	2, 3	Figure 5, Supp. Figure S2B
	fibronectin	*fn1*	ECM assembly, cell motility, adhesion, wound healing	Extracellular space, ECM organization, Acute-phase response	1, 2	Figure 4B, Figure 5
	biglycan-like	*bgn*	Collagen fiber assembly, ECM organization, matrix mineralization	Extracellular space, ECM organization, Collagen-containing ECM	1	Figure 4B
2. Immunity and defense (Not including antiviral response)	cathelicidin	*camp*	Antibacterial activity	Defense response to bacterium, Antimicrobial humoral immune response mediated by antimicrobial peptide	2	Figure 5
	beta-galactoside-binding lectin	*lgals1*	Regulating apoptosis, cell proliferation and cell differentiation	Extracellular space, Regulation of immune system process	1	Figure 4B
	CD59 glycoprotein	*cd59*	Complement MAC inhibition	Regulation of immune system process, Cell surface receptor signaling pathway	1	Figure 4B
	CD83	*cd83*	Antigen presentation and lymphocyte activation	Regulation of immune system process, Leukocyte activation, Positive regulation of immune system process	1	Figure 4B
	complement C1q tumor necrosis factor-related protein 3	*c1qtnf3*	Regulatory role in immune system	^$^ Negative regulation of inflammatory response, Positive regulation of cytokine secretion, Immune system process	1	Figure 4A
	complement C1q-like protein 2	*c1ql2*	Regulate excitatory synapses	^$^ Extracellular region, Immune system process	4	Supp. Figure S3A
	C-reactive protein	*crp*	Inflammation, acute phase response, defense	Leukocyte mediated immunity, Regulation of immune system process, Defense response	1	Figure 4B
	coagulation factor V-like	*f5*	Central regulator of hemostasis	Platelet alpha granule lumen, Platelet degranulation	2	Figure 5
	putative defense protein Hdd11	*hdd11*	Putative antimicrobial activity	^$^ Defense response to other organism, Response to stimulus, Innate immune response	1	Figure 4B
	CCAAT/enhancer-binding protein beta-like	*cebpb*	Transcription factor for immune and inflammatory genes	Defense response to bacterium, Regulation of cytokine biosynthetic process, Acute-phase response	2	Figure 5
3. Chemotaxis and signaling	C-X-C motif chemokine 2/MIP2-aplha/permeability factor 2-like	*cxcl2*	Hematoregulatory chemokine	Myeloid leukocyte migration, Neutrophil chemotaxis, Chemokine-mediated signaling pathway	2	Figure 5
	leukocyte cell derived chemotaxin 2	*lect2*	Chemotactic activity, chondrocyte proliferation	^$^ Response to stimulus, Immune system process	2	Figure 5
	C-C motif chemokine 17-like	*ccl17*	T-lymphocytes trafficking and activation	Response to cytokine, Cellular response to cytokine stimulus	3	Figure 2B
	chemokine receptor 4-like	*ccrl1*	Regulator of chemotaxis	^$^ Response to stimulus, Immune system process	1	Figure 4B
	C-X-C motif chemokine 11-like	*cxcl11*	Chemotactic for activated T-cells, neutrophils or monocytes	Myeloid leukocyte migration, Neutrophil chemotaxis, Chemokine-mediated signaling pathway	2	Figure 5
4. Antiviral response	radical S-adenosyl methionine domain-containing protein 2, alias (viperin)	*rsad2*	Antiviral	Immune system, Regulation of immune system process	1	Figure 4B
	interferon a3	ifna^*^	Antiviral	Immune system process, Response to stimulus	1	Figure 4A
5. Antioxidant activity and redox homeostasis	thioredoxin	*txn*	Redox homeostasis	^$^ Antioxidant activity, Response to oxygen levels, Response to stress	2, 3	Figure 5, Supp. Figure S2B
	glutathione S-transferase A	*gsta*	Redox homeostasis	^$^ Response to stress/chemical	1	Figure 4B
	glutathione peroxidase 7	*gpx7*	Redox homeostasis	^$^ Antioxidant activity, Response to stimulus	1	Figure 4B
	redox-regulatory protein fam213a (peroxiredoxin-like 2A)	*prxl2a*	Redox homeostasis	^$^ Antioxidant activity, Regulation of immune system process	1	Figure 4B
	peroxiredoxin 1	*prdx1*	Redox homeostasis	^$^ Removal of superoxide radicals, Response to oxidative stress	3	Figure 2B
	neutrophil cytosol factor 2-like isoform X2	*ncf2*	NADPH oxidase, redox homeostasis	^$^ Superoxide-generating NADPH oxidase activator activity, Respiratory burst	1	Figure 4B

^1^ Based on functional classes, candidate genes are categorized under five themes for discussion purposes. ^2^ Name(s) or alias(es) obtained from annotation. Refer the Supplementary File S1 for additional details. ^3^ Official gene symbols (version 1) are based on multiple annotations and the majority of them are represented in HUGO Gene Nomenclature Committee (HGNC) (https://www.genenames.org/) and/or GeneCards (https://www.genecards.org/) databases. ^4^ General overview of the function of each candidate gene extracted from the UniProtKB database (https://www.uniprot.org/uniprot/). ^5^ Selected enriched GO terms associated with a GOI obtained from enrichment analyses. Additional details are available in Supplementary files. ^6^ In which segment (refer to Figure 2) of the microarray data list, the majority of the DEPs representing this transcript is present. ^#^ This was not identified by the microarray in this study. ^*^ Putative gene symbol of *ifna* (LOC106607463). ^$^ One or more terms were obtained from UniProtKB/Blast2GO analyses.

## Supplementary Materials

Supplementary Materials can be found at https://www.mdpi.com/1422-0067/21/7/2417/s1.
