# Supplementary material for: Transcriptomic Profiling in Fins of Atlantic Salmon Parasitized with Sea Lice: Evidence for an Early Imbalance Between Chalimus-Induced Immunomodulation and the Host’s Defense Response
Source: Int J Mol Sci. 2020 Mar 31;21(7):2417. doi: 10.3390/ijms21072417 (PMC7177938; doi:10.3390/ijms21072417)
Supplement: Supplementary file 1 [file ijms-21-02417-s001.zip › ijms-721524-final-supplementary-revised/Supplementary_Fig/Suppl_Figs_except_Sup_Fig_6.docx]

Article

Transcriptomic Profiling in Fins of Atlantic Salmon Parasitized with Sea Lice: Evidence for an Early Imbalance Between Chalimus-induced Immunomodulation and the Host’s Defense Response

Navaneethaiyer Umasuthan ^1^*, Xi Xue ^1†^, Albert Caballero-Solares ^1†^, Surendra Kumar ^1^, Jillian D. Westcott ^2^, Zhiyu Chen ^2^, Mark D. Fast ^3^, Stanko Skugor ^4^, Barbara F. Nowak ^5^, Richard G. Taylor ^6^ and Matthew L. Rise ^1^*

^1^ Department of Ocean Sciences, Memorial University of Newfoundland, St. John's, NL A1C 5S7, Canada; [navaumasuthan@gmail.com](mailto:navaumasuthan@gmail.com) (N.U.); [xi.xue@mun.ca](mailto:xi.xue@mun.ca) (X.X.); [acaballeroso@mun.ca](mailto:acaballeroso@mun.ca) (A.C.S); [surendrak@mun.ca](mailto:surendrak@mun.ca) (S.K.); [mrise@mun.ca](mailto:mrise@mun.ca) (M.L.R.)

^2^ Fisheries and Marine Institute, Memorial University of Newfoundland, P.O. Box 4920, St. John's, NL A1C 5R3, Canada; [jillian.westcott@mi.mun.ca](file:///C:\Users\unavaneethai\Downloads\ijms-21-02417-s001%20(1)\Supplementary_Materials\Supplementary_Fig\Jillian.Westcott@mi.mun.ca) (J.D.W.); [zc5118@mun.ca](file:///C:\Users\unavaneethai\Downloads\ijms-21-02417-s001%20(1)\Supplementary_Materials\Supplementary_Fig\zc5118@mun.ca) (Z.C.)

^3^ Department of Pathology and Microbiology, Atlantic Veterinary College, University of Prince Edward Island, Charlottetown, PEI, Canada; [mfast@upei.ca](mailto:mfast@upei.ca)

^4^ Cargill Aqua Nutrition, Cargill, Sea Lice Research Center (SLRC), Hanaveien 17, 4327 Sandnes, Norway; [stanko_skugor@cargill.com](file:///C:\Users\unavaneethai\Downloads\ijms-21-02417-s001%20(1)\Supplementary_Materials\Supplementary_Fig\Stanko_Skugor@cargill.com)

^5^ Institute of Marine and Antarctic Studies, University of Tasmania, Locked Bag 1370, Launceston 7250, Tasmania, Australia; [b.nowak@utas.edu.au](mailto:b.nowak@utas.edu.au)

^6^ Cargill Animal Nutrition, 10383 165th Avenue NW, Elk River, MN 55330, USA; [richard_taylor@cargill.com](mailto:richard_taylor@cargill.com)

***** Correspondence: [navaumasuthan@gmail.com](mailto:navaumasuthan@gmail.com) (N.U.); [mrise@mun.ca](mailto:mrise@mun.ca) (M.L.R.);

Tel.: +1-709-864-3247 (N.U.); +1-709-864-7478 (M.L.R.)

† These authors contributed equally to this work.

**Supplementary Figures**

**
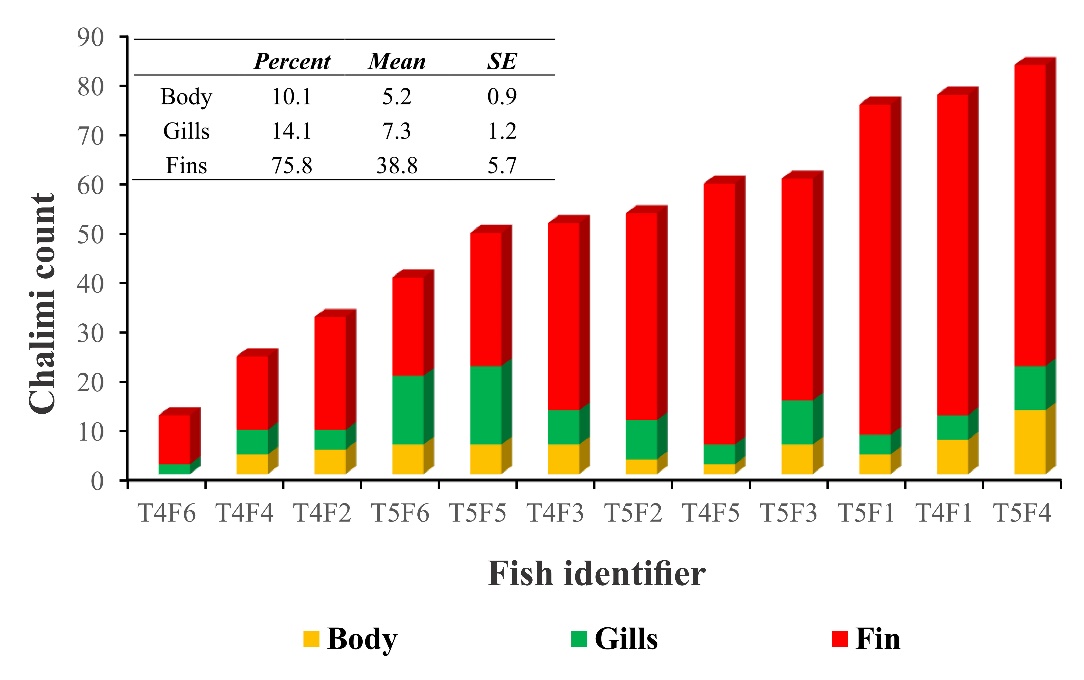
**

**Supplementary Figure S1.** Distribution of *L. salmonis* chalimi in three different regions of the Atlantic salmon body. Data show the chalimi count determined on 12 fish from two individual tanks. Sea lice burden was quantified at 8 days post-infection (dpi), and body regions of salmon, i.e., body, gills, and fins are shown in yellow, green, and red, respectively. Inset table shows percent and mean chalimi count with SE for each region. The average parasite burden in the entire fish was determined to be 51 ± 6.3 (mean ± SE). One-way ANOVA followed by Tukey's multiple comparisons test indicated that lice load in fins was significantly (*p* < 0.0001) different from gills and the rest of the body.


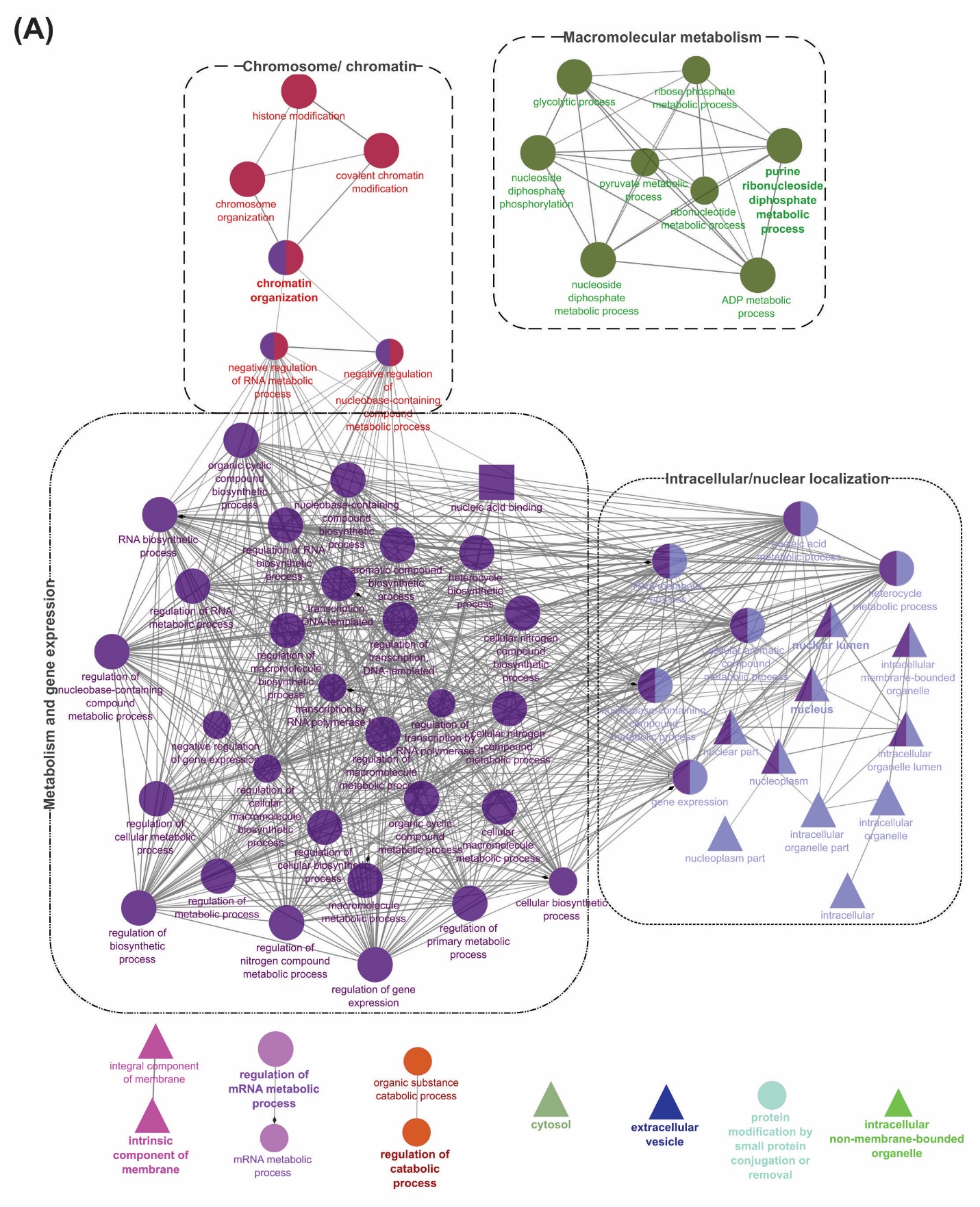


**Supplementary Figure S2.** See next page.


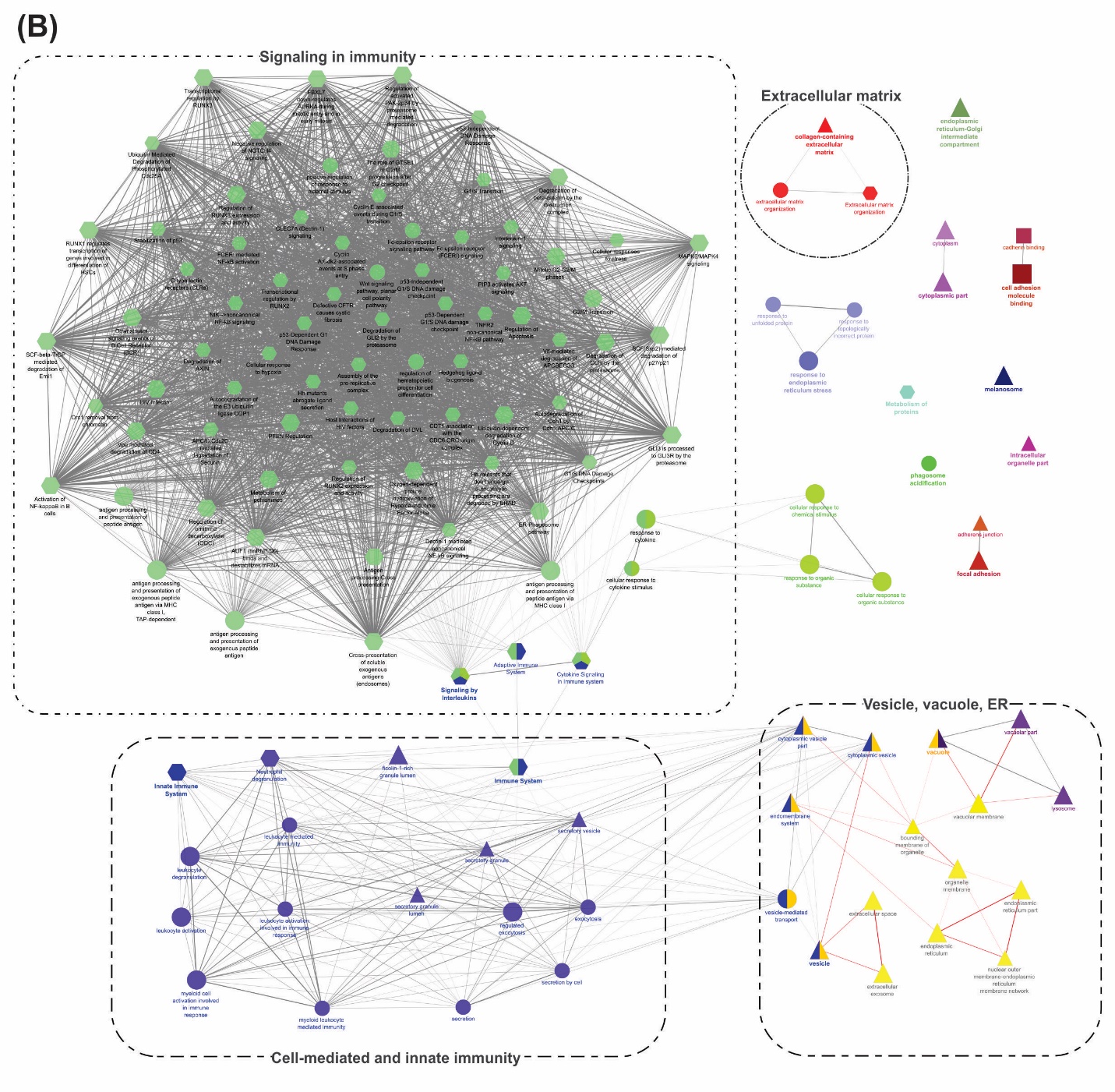


**Supplementary Figure S2.** Gene-Ontology (GO) term enrichment and pathway term network analysis of DEGs unique for ADJ vs. PRE list (4259 DEPs). (A) Up-regulated DEGs (3166 DEPs). (B) Down-regulated DEGs (1093 DEPs). GO term enrichment analysis was performed by the ClueGO [1] plugin in Cytoscape [2]. Two databases were used including GO (BP, MF and CC) and Reactome pathways for retrieving associated GO terms. Only the networks and pathways with p < 0.05 are illustrated. Functionally grouped networks with terms as nodes linked by edges based on their kappa score level (≥0.4) are shown. Related GO terms are grouped and illustrated with a distinct color and labeled with the same color. The node size represents the significance of term enrichment. Functionally related groups partially overlap. When a particular GO term is shared by two/more different GO cluster groups, the node is shown by multiple colors. The shape of the nodes indicates the source of the database from where a term was retrieved (ellipse, GO_BP; rectangle, GO_MF; triangle, GO_CC; Reactome, hexagon). The thickness of edges indicates the kappa score (strength of intra-connectivity between cluster groups). Related clusters are shown together within a dotted border and labeled with broad themes for discussion purposes. Refer to the Supplementary Files S10 and S11 for additional details.

**
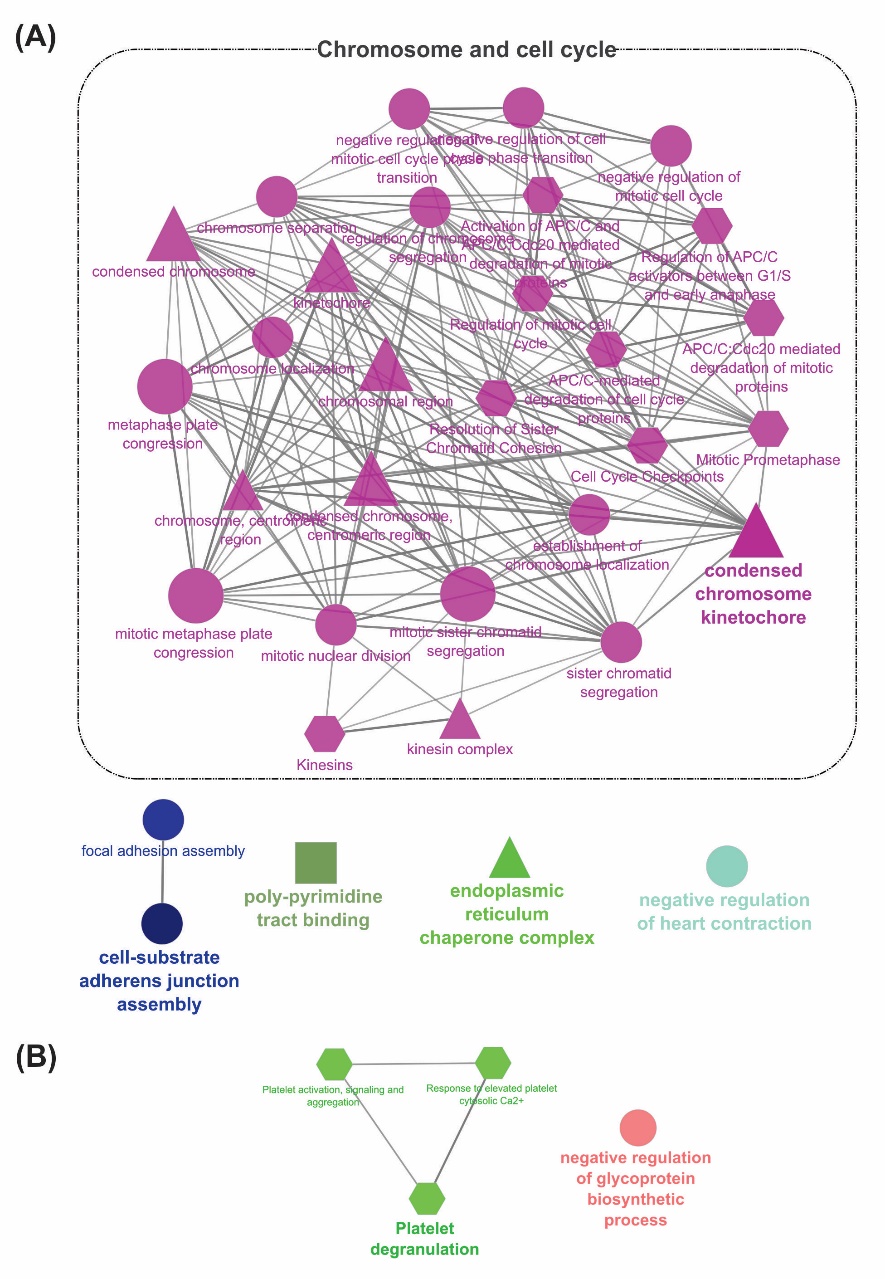
**

**Supplementary Figure S3.** Gene-Ontology (GO) term enrichment and pathway term network analysis of DEGs unique for ATT vs. PRE list (296 DEPs). (A) Up-regulated DEGs (113 DEPs). (B) Down-regulated DEGs (183 DEPs). GO term enrichment analysis was performed by the ClueGO [1] plugin in Cytoscape [2]. Two databases were used including GO (BP, MF and CC) and Reactome pathways for retrieving associated GO terms. Only the networks and pathways with p < 0.05 are illustrated. Functionally grouped networks with terms as nodes linked by edges based on their kappa score level (≥0.4) are shown. Related GO terms are grouped and illustrated with distinct color and labeled with the same color. The node size represents the significance of term enrichment. Functionally related groups partially overlap. When a particular GO term is shared by two/more different GO cluster groups, the node is shown by multiple colors. The shape of the nodes indicates the source of the database from where a term was retrieved (ellipse, GO_BP; rectangle, GO_MF; triangle, GO_CC; Reactome, hexagon). The thickness of edges indicates the kappa score (strength of intra-connectivity between cluster groups). Related clusters are shown together within a dotted border and labeled with broad themes for discussion purposes. Refer to the Supplementary Files S12 and S13 for additional details.

**
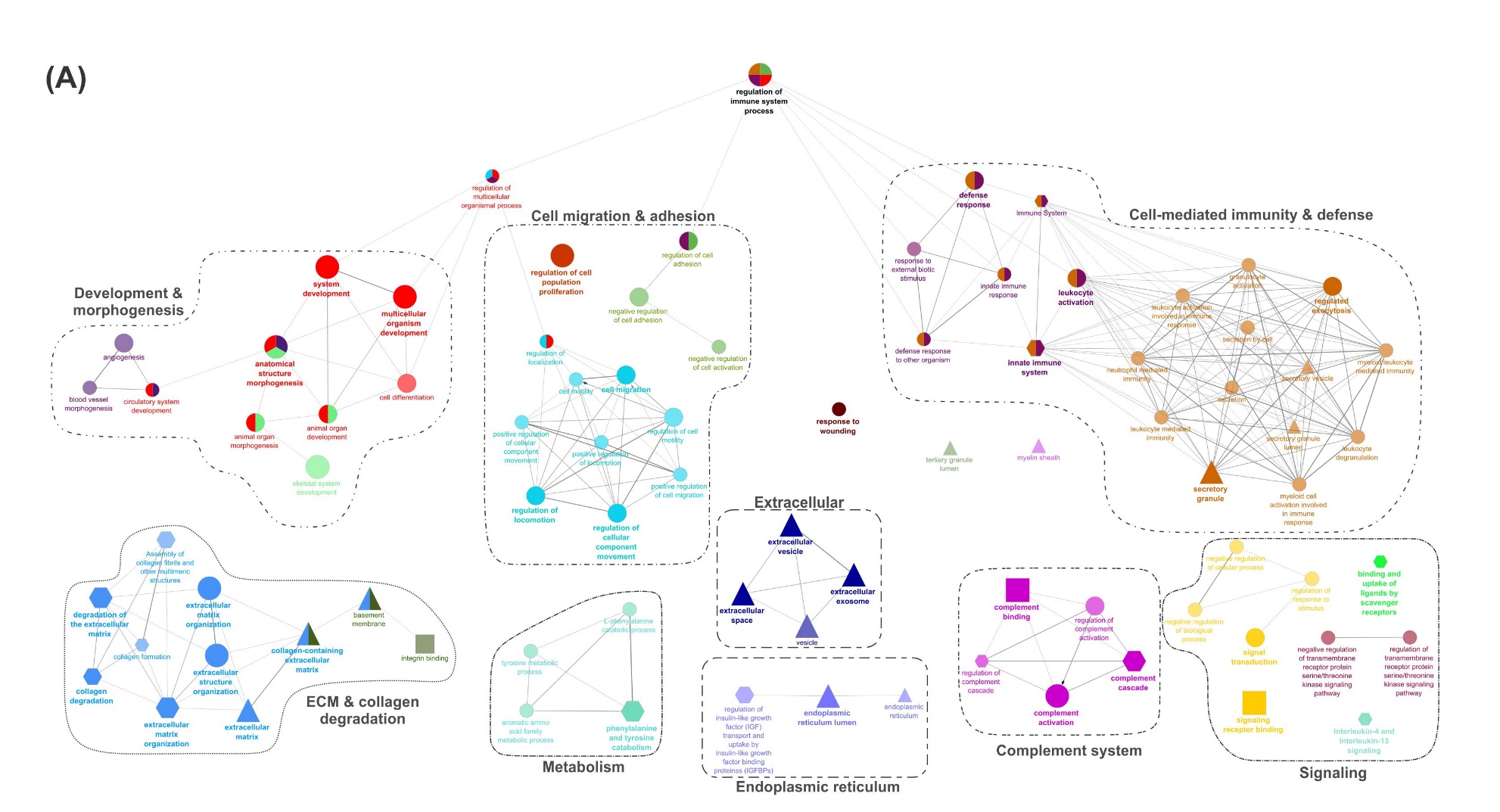
**

**Supplementary Figure S4.** See next page.

**
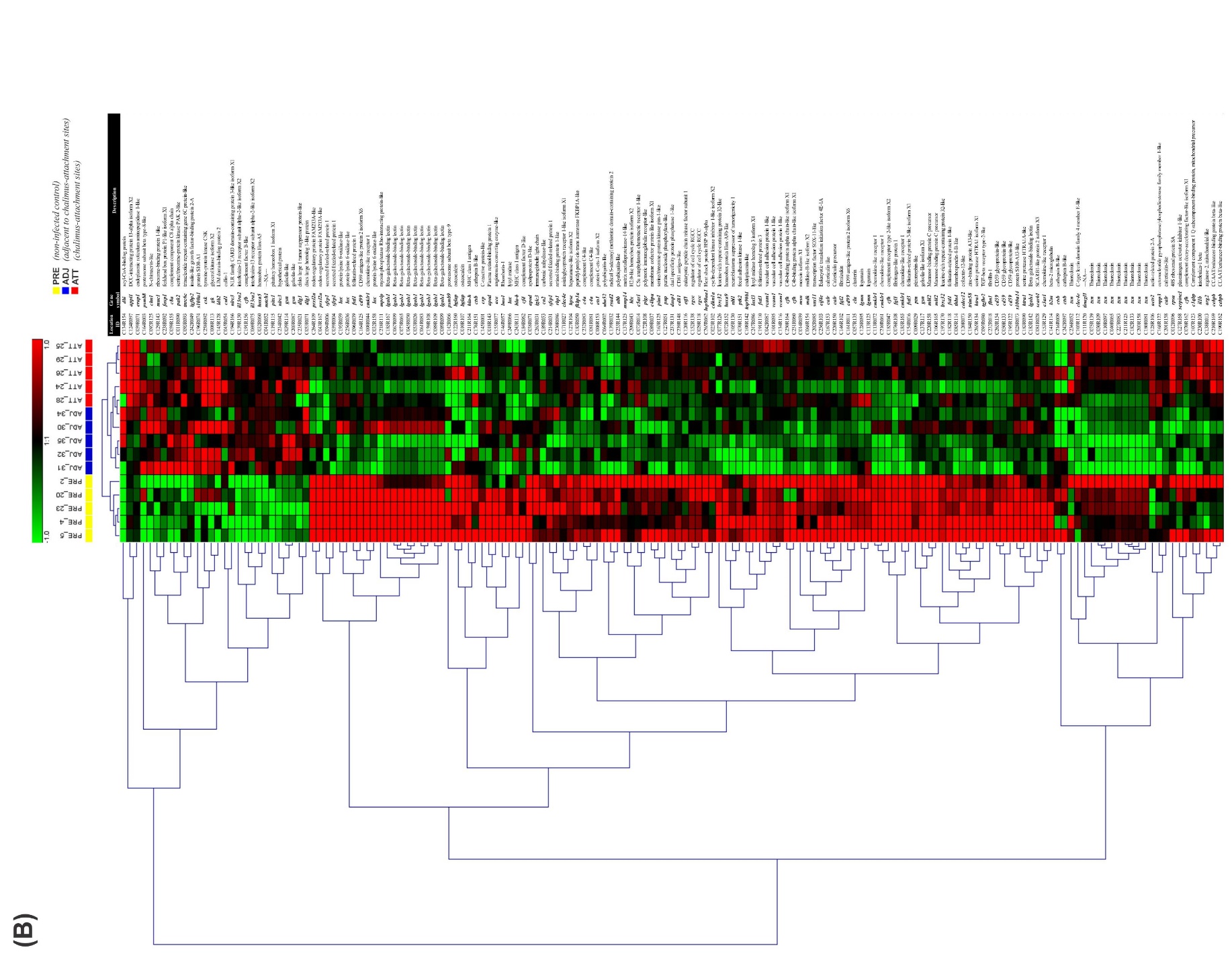
**

**Supplementary Figure S4.** (A) Gene-Ontology (GO) term enrichment and pathway term network analysis of unique DEPs (fold-change>|2|) resulting from all possible comparisons and (B) heatmap illustration and hierarchical clustering analyses of DEPs representing ‘regulation of immune system process’ (GO:0002682) that appeared to be the master node in ClueGO analyses. (A) GO term enrichment analysis was performed by the ClueGO [1] plugin in Cytoscape [2]. Two databases were used including GO (BP, MF and CC) and Reactome pathways for retrieving associated GO terms. Only the networks and pathways with p < 0.05 are illustrated. Functionally grouped networks with terms as nodes linked by edges based on their kappa score level (≥0.4) are shown. Related-GO terms are grouped and illustrated with distinct color and labeled with the same color. The node size represents the significance of term enrichment. Functionally related groups partially overlap. When a particular GO term is shared by two/more different GO cluster groups, the node is shown by multiple colors. The shape of the nodes indicates the source of the database from where a term was retrieved (ellipse, GO_BP; triangle, GO_CC; GO_MF, square; Reactome, hexagon). The thickness of edges indicates the kappa score (strength of intra-connectivity between cluster groups). Related clusters are shown together within a dotted border and labeled with broad themes for discussion purposes. Refer to the Supplementary File S14 for details. (B) GO:0002682 connected three main immune relevant clusters (i.e., ‘development and morphogenesis’, ‘cell migration and adhesion’ and ‘cell-mediated immunity and defense’) in our ClueGO analyses of entire DEPs with fold-change>|2| (Supplementary Figure S4 and Supplementary File S14) and subjected to heatmap construction. Rows and columns represent the log2 fold-changes of different transcript expression levels (163 DEPs/ 110 DEGs) and individual fish from the lice-infection groups (colored boxes), respectively. Genes were median-centered and clustered using Pearson correlation and complete linkage hierarchical clustering. The colored boxes below the top legend represent individual fish from the lice-infection groups.

**
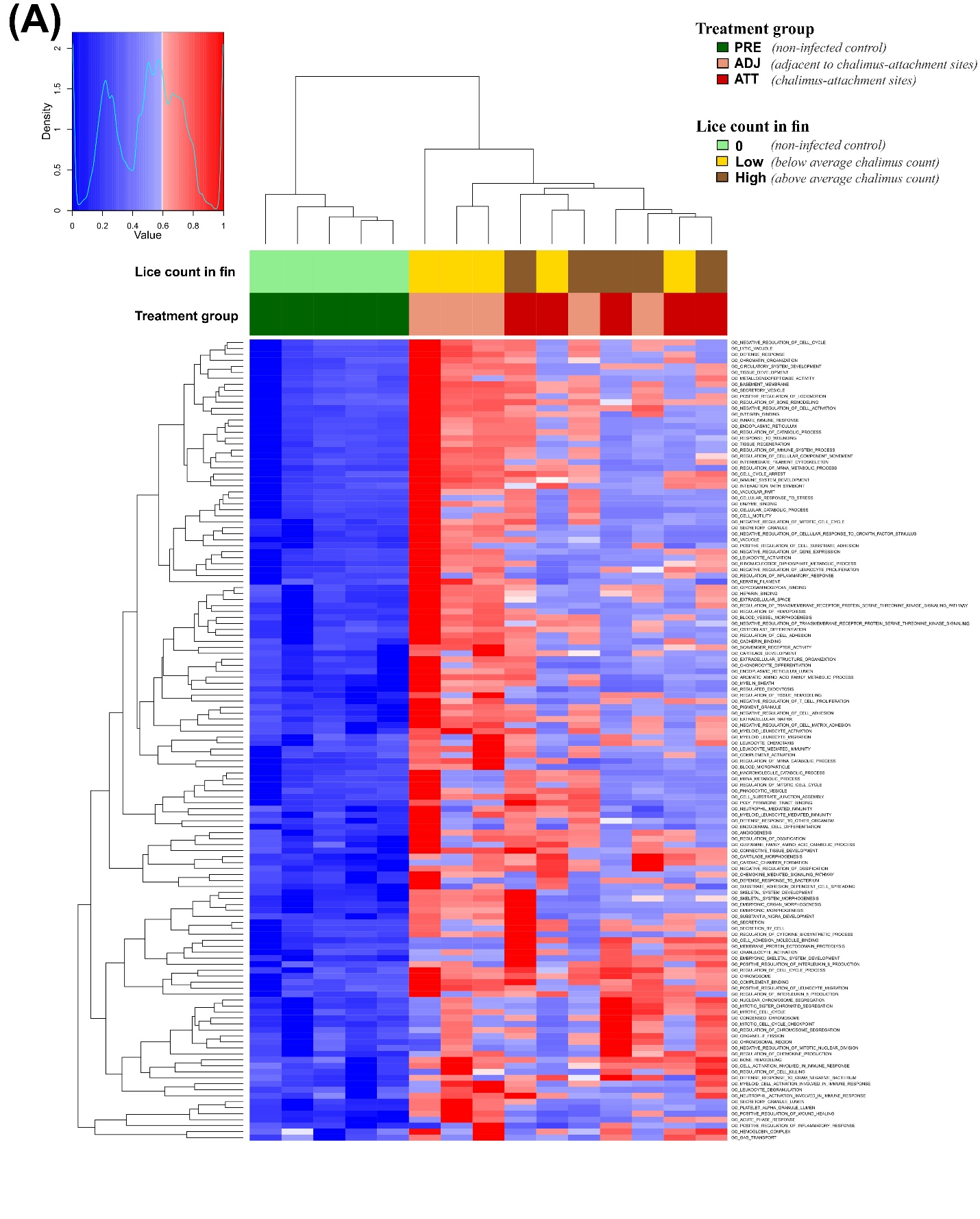
**

**Supplementary Figure S5.** See next page.

**
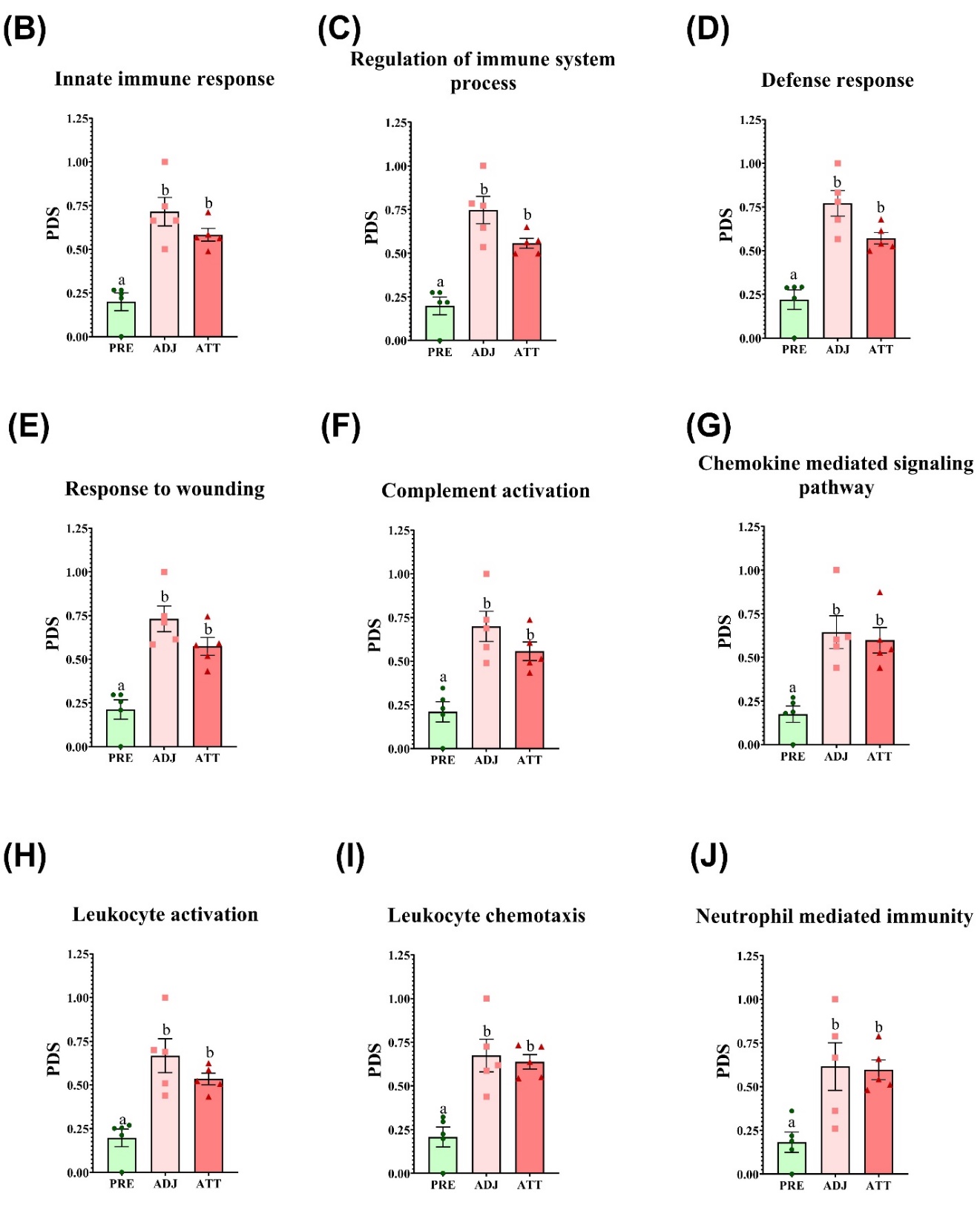
**

**Supplementary Figure S5.** Pathway deregulation scores (PDS) for enriched GO terms. (A) PDS-based clustering analysis and heatmap for 134 enriched GO pathways from ClueGO data. All the enriched GO terms resulting from ClueGO analyses (n=249) were matched with MSigDB, and 134 annotated enriched pathways and their expression data were used in calculating PDS. Unsupervised clustering on PDS of 15 fish from three treatment groups (PRE, green; ADJ, light red; ATT, dark red) is shown. Chalimus count in the fin is indicated as 0 (PRE; light green), low (yellow) and high (brown), where low and high levels were below and above the average chalimus count, respectively. GO pathway description from MSigDB is shown next to each block. (B-J) PDS of selected immune-relevant GO terms across different treatments (PRE, ADJ and ATT). Different letters above bars represent significant differences between groups [one-way ANOVA, Tukey’s (homogeneity of variances among groups) post hoc test, p < 0.05].

**Supplementary Figure S6.** containing all the high-resolution heatmaps on different sheets in a separate excel file, named “**Suppl. Fig. S6**”.

**
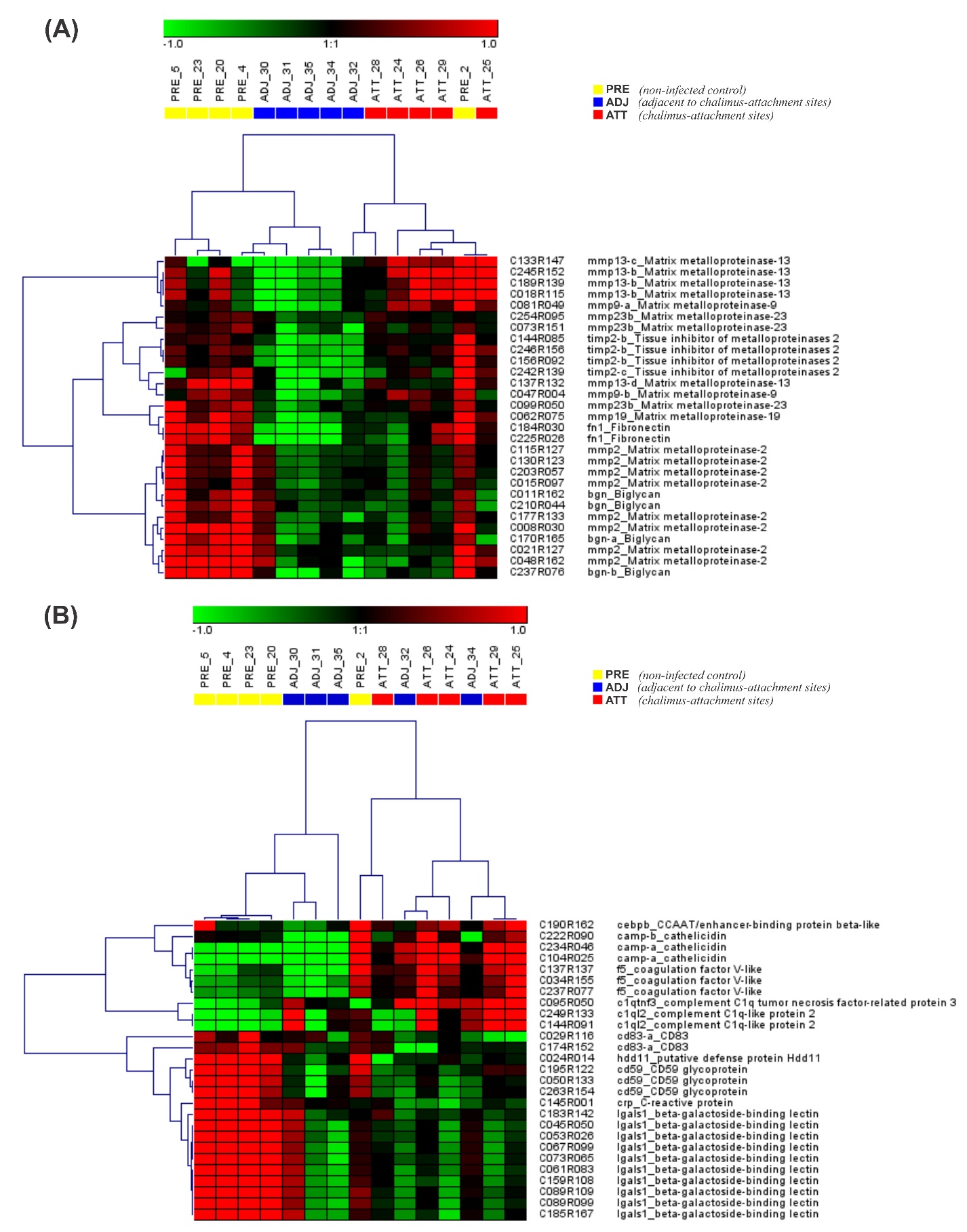
**

**Supplementary Figure S7.** See next page

**
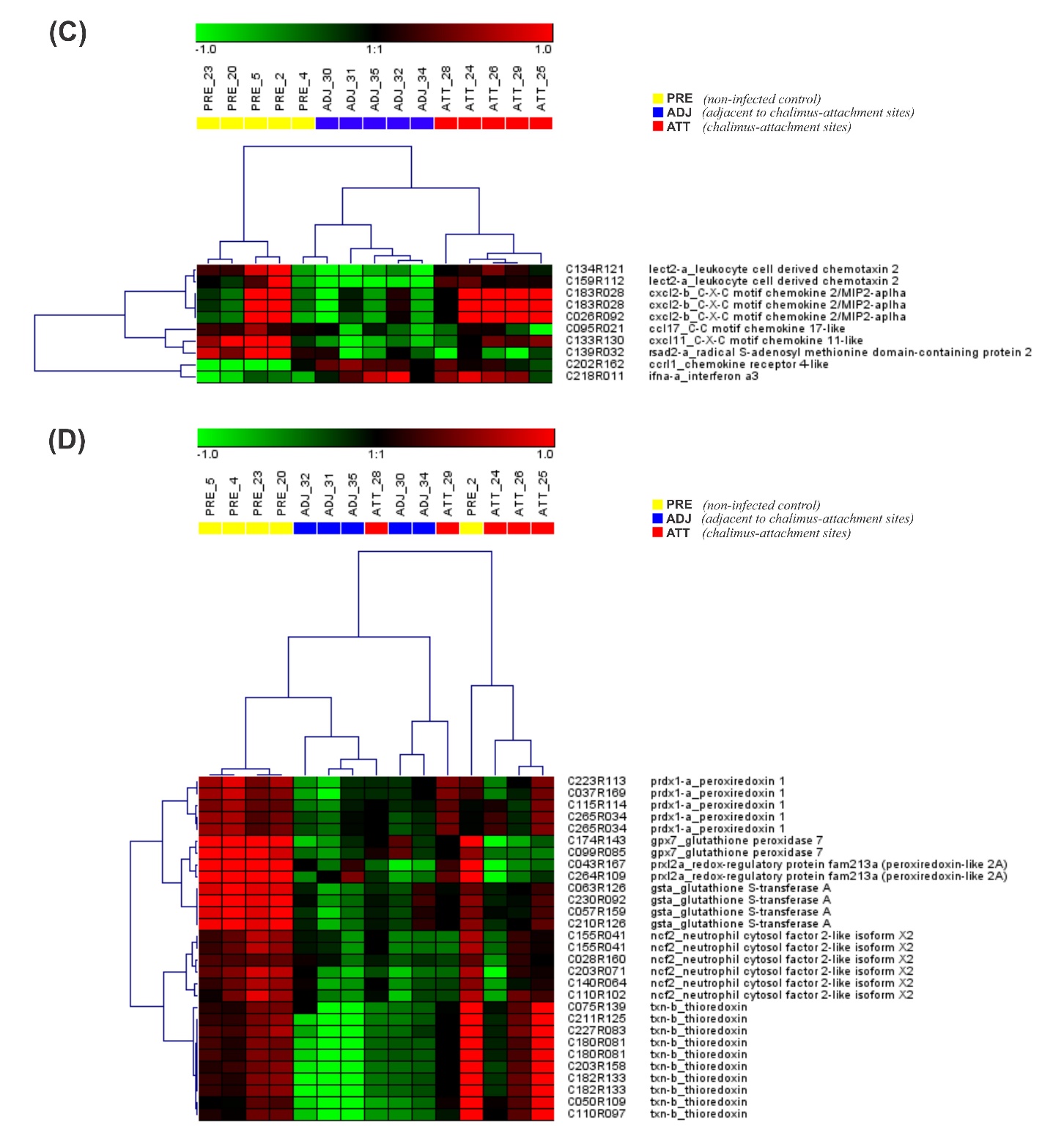
**

**Supplementary Figure S7.** Heatmap illustration and hierarchical clustering analyses of chalimus-responsive DEGs associated with different functional themes and chosen for QPCR confirmation. Rows and columns represent the log2 fold-changes of different transcript expression levels and individual fish from the lice-infection groups (colored boxes), respectively. Genes were median-centered and clustered using Pearson correlation and complete linkage hierarchical clustering based on microarray data. These QPCR targets were categorized under five themes based on their functions (refer to Table 3). (A) Theme 1 (ECM, tissue repair/remodeling and wound healing), (B) Theme 2 (immunity and defense), (C) Theme 3 (chemotaxis and signaling) and Theme 4 (antiviral response) and (D) Theme 5 (antioxidant activity and redox balance).

References

1. Bindea, G.; Mlecnik, B.; Hackl, H.; Charoentong, P.; Tosolini, M.; Kirilovsky, A.; Fridman, W.-H.; Pagès, F.; Trajanoski, Z.; Galon, J., ClueGO: a Cytoscape plug-in to decipher functionally grouped gene ontology and pathway annotation networks. Bioinformatics 2009, 25, (8), 1091-1093.

2. Shannon, P.; Markiel, A.; Ozier, O.; Baliga, N. S.; Wang, J. T.; Ramage, D.; Amin, N.; Schwikowski, B.; Ideker, T., Cytoscape: a software environment for integrated models of biomolecular interaction networks. Genome Res. 2003, 13, (11), 2498-2504.
